# Bayesian Methods Incorporating Baseline Covariates for Borrowing Historical Control Data in Randomized Clinical Trials: A Neutral Comparison Study

**DOI:** 10.1002/sim.70785

**Published:** 2026-10-22

**Authors:** David Jesse, Diane Uschner, Markus C. Elze, Tim Friede, Francois Mercier

**Affiliations:** ^1^ F. Hoffmann‐La Roche AG Basel Switzerland; ^2^ Department of Medical Statistics University Medical Center Göttingen Göttingen Germany; ^3^ DZKJ (German Center for Child and Adolescent Health) Göttingen Germany

**Keywords:** baseline covariates, Bayesian methods, dynamic borrowing, historical control, neutral comparison, randomized clinical trial

## Abstract

The idea of leveraging historical control data in randomized clinical trials (RCTs) has gained a lot of attention, in particular for rare disease and pediatric indications where conducting traditional and fully powered RCTs can be challenging. In this regard, Bayesian dynamic borrowing (BDB) methods, which adapt the amount of borrowing based on the outcome heterogeneity between the current and historical data, are well established. In recent years, an increasing number of related methods have been published that combine the BDB framework with approaches that account for baseline covariates. The underlying rationale is that some of the heterogeneity may be explained systematically by variations in the distributions of these characteristics. While many such methods are now available, direct comparisons of them are scarce and there is no consensus on which one can be considered the state of the art. We address this issue by conducting a neutral comparison of three methods of this class, namely the propensity score weighted commensurate prior, propensity score weighted multi‐source exchangeability models, and Bayesian additive regression trees. We assess their performance for continuous outcomes through extensive simulations and further evaluate them using two examples based on real RCT data. Our study reveals potential issues with some of these approaches and provides recommendations for choosing a particular method for real RCTs.

## 1 Introduction

Despite their scientific rigor, implementing randomized clinical trials (RCTs) in rare diseases or pediatric indications can be challenging due to large required sample sizes, long durations, and high operational costs. To address these hurdles, the idea of incorporating historical control arm data from previous RCTs in the same indication into the analysis of a new trial has gained interest from health authorities and clinical trial sponsors. This idea is called (*dynamic*) *borrowing* [1] and has traditionally been approached in a Bayesian framework by constructing informative prior distributions from the historical data to be applied in the new trial. The power prior [2] and the meta‐analytic predictive prior [3] are two early and popular examples of such methods. It is well known, however, that a strict frequentist Type I error control is not possible when aiming to gain power by leveraging external information [4]. Still, the risk of drawing false conclusions due to a prior‐data conflict, that is, the situation when the current and historical data are in disagreement, can be attenuated by using extensions of aforementioned priors that anticipate this kind of situation [5, 6]. Another recent idea to further mitigate these risks is to also take into account prognostic baseline covariate information. The rationale behind this is that discrepancies in the outcome distributions between the current and historical trial data may be caused by variations in prognostic baseline characteristics that can be controlled for. Such an approach also aligns with the Pocock criteria for integrating historical data in RCTs which, among others, demand that “(t)he distribution of important patient characteristics in the group should be comparable with those in the new trial” [7]. For this reason, new borrowing methods have been developed that suggest to integrate ideas from the causal inference literature into the Bayesian dynamic borrowing (BDB) framework. *Propensity score integrated priors* [8] form the largest group of these methods. They are characterized by a two‐step procedure that first applies some kind of propensity score adjustment to the historical data [9] and, after that, utilizes a BDB prior. They claim to make the borrowing more robust and efficient by, at least partially, relating the discrepancy (agreement) in the outcome distributions to differences (similarities) in baseline characteristics and making corresponding adjustments. Therefore, the strength of these methods can be characterized by their ability to handle observed confounding induced by different distributions of prognostic baseline covariates while also accounting for unobserved heterogeneity through the use of Bayesian methods. A large number of such approaches have recently been proposed in the literature and while these methodological developments are desirable, they also pose new challenges. For the planning of a specific clinical trial, the question is raised which of the many available methods to choose. For instance, trial sponsors may be particularly interested in the possible efficiency gains that can be expected by applying the different methods. On the other hand, health authorities pay special attention to the robustness of these methods in cases of discrepancies between the current and historical data. Making a decision for one approach is not trivial, however, since the performance evaluations of these methods, commonly conducted by the authors via simulation studies, tend to be over‐optimistic in favor of the new proposal [10]. For instance, different data‐generating mechanisms with different underlying assumptions can be used for the simulation or the selection of comparator methods can differ between studies. As a consequence, there is often no consensus on what method(s) can be considered the leading benchmark in specific applications, leaving researchers with a plethora of available approaches and no guidance on which one to choose. Neutral comparison studies [11] can address this issue: “(T)hey do not aim to demonstrate the superiority of a particular method and are thus not designed in a way that may increase the probability to observe incorrectly this superiority” [10]. Building on these arguments, the aim of the present paper is to systematically compare different covariate‐adjusted BDB methods for leveraging historical control data in RCTs for continuous endpoints. Specifically, we selected three recently proposed methods with different underlying modeling mechanisms for this comparison. These include the propensity score weighted commensurate prior [8], propensity score weighted multi‐source exchangeability models [12], and Bayesian additive regression trees [13]. The remainder of this paper is organized as follows: We begin by establishing the essential notation and formally defining the problem of interest. Further, we briefly discuss some potential problems when seeking to incorporate historical control data into RCTs (Section 2). We then introduce the three methods under investigation and explain their respective approach to estimating the treatment effect while leveraging external information (Section 3). After that we compare these methods in a comprehensive simulation study and discuss the results (Section 4). To augment these insights, we also apply the methods to two illustrative examples based on real clinical trial data (Section 7). Finally, we conclude our findings and discuss some ideas for future research (Section 8).

## 2 Notation, Estimands & Assumptions

We consider the setting of an RCT with outcome variable, Y, that is the clinical endpoint of interest. Subjects enrolled in the RCT are randomly assigned to receive a binary treatment, A, where A=1 indicates assignment to the experimental treatment group and A=0 assignment to the control group. Additionally, a vector of baseline covariates, X, is recorded for each subject. Our setting differs from traditional RCTs as we also aim to incorporate control group data from previous RCTs in the same indication to potentially improve efficiency and conserve resources in the new trial. To this end, we introduce a study indicator $S\in \left\{0, 1, 2,\ldots ,S^{\prime }\right\}$, where S=0 marks participation in the current RCT, while $S=1,2,\ldots ,S^{\prime }$ specifies that the data come from one of the respective historical RCTs. We denote the observed data from the s‐th trial as $D_{s}=\left\{\left(y_{i},a_{i},x_{i}\right):i=1,2,\ldots ,n_{s}\right\}$. Based on this, the pooled data is defined as $D={⋃}_{s=0}^{S^{\prime }}D_{s}$. To define the target estimand when borrowing historical control data, we follow recent suggestions to adapt the potential outcomes framework [14] to this kind of setting [15, 16]. Correspondingly, we denote by $Y^{\left(A\right)}$ the potential outcome under treatment A. Precisely, $Y^{\left(1\right)}$ represents the potential outcome had the experimental treatment been received, and $Y^{\left(0\right)}$ represents the potential outcome had the control treatment been given instead (e.g., placebo or standard of care). In practice, for an individual i, we only observe $Y_{i}=A_{i}Y_{i}^{\left(1\right)}+\left(1-A_{i}\right)Y_{i}^{\left(0\right)}$, that is the potential outcome under the treatment that has actually been received. An individual causal effect can be defined by considering a contrast of the potential outcomes for subject i. In this paper, we consider differences $Y_{i}^{\left(1\right)}-Y_{i}^{\left(0\right)}$. Based on these considerations, we first define the conditional average treatment effect (CATE):

$$\Delta_{c}\left(x\right)≔E\left[Y^{\left(1\right)}-Y^{\left(0\right)}|X=x\right]$$

As explicitly shown here, the CATE is the average treatment effect dependent on a specific configuration of the baseline covariates x. In RCTs, however, we primarily aim to demonstrate a clinically relevant effect for the entire population regardless of their baseline characteristics. Furthermore, when incorporating historical data, we need to make explicit that we are interested in the treatment effect in the current RCT. This leads us to the average treatment effect (ATE) in the current trial population, which we define as:

$$\Delta ≔E\left[Y^{\left(1\right)}-Y^{\left(0\right)}|S=0\right]=E_{X|S=0}\left[\Delta_{c}\left(x\right)|S=0\right]$$

Hence, this effect can be derived by averaging the conditional effects of the current trial participants over the covariate distribution of that same population. For convenience, we refer to this effect definition simply as the ATE in the remainder of this paper, without further emphasizing that this relates to the current trial.

In the causal inference literature, it is common practice to establish identification assumptions under which, in theory, it is possible to estimate the target estimand from the observed data [17]. Among others, Zhou et al. have transferred this idea to the problem of estimating treatment effects when incorporating historical control data into a new RCT. We adopt this idea in the following but want to point out that we do not expect all of these assumptions to be ever satisfied in practical applications. Rather, we think that explicitly stating these assumptions is valuable for discussing potential sources of heterogeneity between the current and historical data sources and how severe their impact could be on the analysis.

(*A1*) *No direct effect of trial participation*.

The potential outcomes under the control treatment are independent of trial participation [16]. Formally, $\left[Y\left(0\right)|S=0\right]=Y\left(0\right)\forall S\in \left\{0,1,\ldots ,S^{\prime }\right\}$. This assumes that study conditions do not influence outcomes of patients in the control group.

(*A2*) *Stable unit treatment value assumption* (*SUTVA*).

This encompasses two sub‐assumptions: consistency and no interference. Consistency implies that an individual's observed outcome equals their potential outcome under the received treatment: $Y_{i}=Y_{i}^{\left(A_{i}\right)}$. In our context of borrowing historical control data, this can be refined as $Y_{i}=Y_{i}^{\left(A_{i}\right)}\;\text{for}\;\left(y_{i},a_{i},x_{i}\right)\in D_{0}$ and $Y_{i}=Y_{i}^{\left(0\right)}\;\text{for}\;\left(y_{i},a_{i},x_{i}\right)\notin D_{0}$. No interference means that the individual potential outcomes are mutually independent.

(*A3*) *Internal validity of the trial*.

Within the current RCT, treatment assignment is random for all subjects: $\left(Y_{i}^{\left(1\right)},Y_{i}^{\left(0\right)}\right)\;⊥\;A_{i}\;\text{for}\;A_{i}\in \left\{0,1\right\}\;\text{and}\;i\in D_{0}$. Additionally, there is a non‐zero probability of assignment to either treatment group: $0<\mathrm{Pr}\left(A_{i}=1|S_{i}=0\right)<1$.

(*A4*) *Positivity of trial participation*.

Observed covariates, x, must not deterministically predict participation in the ongoing trial: Pr(S=0|x)<1forallx. Hence, for all (historical data) subjects, there is a theoretical possibility of being enrolled in the current trial.

(*A5*) *Conditional ignorability of trial participation*.

Given the covariates, $X_{i}$, the potential outcome under the control treatment is independent of trial participation: $Y_{i}^{\left(0\right)}\;⊥\;S_{i}|X_{i}\;\text{for}\;i\in D$. This implies that all factors influencing both outcomes and study participation are captured in X.

Assumption (A3) refers to the current RCT only and should therefore be fulfilled by design. All other assumptions are, however, subject to violation. (A2) and (A5) can be considered the strongest of all assumptions as they imply that all variation between the current and historical control group can be attributed to prognostic baseline covariates. It is likely, however, that other factors that do not act on the patient‐level and cannot directly be controlled for induce variability in the outcomes, for example, changes in the standard of care over time or different study sites. Either way, knowing and actually recording all confounding variables would still present a substantial challenge. Other assumption violations could also be more subtle. For instance, (A1) may be impacted by differences in study design such as different randomization ratios and associated expectations of patients to receive the novel treatment [18]. We refrain from further elaborating on how each assumption may be violated in practical applications but just want to highlight that, despite the usefulness of different kinds of baseline covariate adjustment, it needs to be expected that a certain amount of between‐trial variation can remain. In this respect, model formulations allowing for this kind of variation such as BDB models have proven useful. Given these considerations, we discuss different covariate‐adjusted BDB methods in the next section. These methods are characterized by combining Bayesian methods for handling unobserved heterogeneity with covariate adjustment techniques from the causal inference literature to account for variations that can be explained systematically, thereby leveraging the strengths of both frameworks.

## 3 Methods

In total, we have selected three covariate‐adjusted BDB methods for our comparison, which we introduce in the following. The selection process was guided by two primary criteria. First, we chose methods that are conceptually distinct, excluding those that were minor variants of others already included. Second, we required that user‐friendly software implementing the respective approach was openly available, either in the form of a software package or structured scripts that could be used to guide our own implementations.

### 3.1 Propensity Score Weighted Commensurate Prior

Propensity score (PS) integrated priors, as mentioned in the introduction, represent a prominent class of methods that merge Bayesian approaches with causal inference techniques for dynamic borrowing [8]. These methods are characterized by a two‐step procedure that begins with a PS adjustment for the combined control arm sample and then applies a BDB prior to derive the posterior distribution of the control group parameter in the current trial that informs the treatment effect. The initial PS adjustment step aims to assess the similarity between the current and historical control group data w.r.t. their baseline covariate distributions. Originating from the causal inference literature, the propensity score is defined as the conditional probability of being assigned to a treatment in observational studies [9]. However, in the context of borrowing historical control data, it takes on a different role, representing the conditional probability of participation in the current trial. For this, we define $\tilde{S}≔1\left(S=0\right)$. Then, the propensity score is defined as

$$e_{i}\left(x_{i}\right)≔\mathrm{Pr}\left({\tilde{S}}_{i}=1|x_{i}\right)$$

In practice, the propensity score must be estimated from the data, commonly through a logistic regression model, although other machine learning algorithms could also be employed. Following this estimation, the augmented control group sample can be adjusted based on the estimated propensity scores using various techniques, including PS‐based stratification, matching, or inverse probability weighting (IPW). The second step of the procedure involves applying an informative BDB prior to the PS‐adjusted sample. A range of options are available for the specification of this prior, including the power prior and its variants [2, 5], the (robust) meta‐analytic predictive prior [3, 6], or the commensurate prior [19]. Having multiple options available for both, the PS adjustment step and the BDB prior, results in a plethora of PS‐integrated prior approaches one can potentially choose from. Some authors have also proposed PS‐integrated priors where the first stage informs the specification of the prior in the second stage, making this pool even larger [20, 21]. For this article, we decided to only include one of these approaches as a representative of this class of methods. Our choice is based on the work by Wang et al. where the authors compared various PS‐integrated priors in a simulation study for binary outcomes. Although some important BDB priors like the robust meta‐analytic predictive (rMAP) prior or the modified power prior were not included in their comparison, to our knowledge this is the most extensive performance comparison of PS‐integrated priors that exists in the literature so far. The authors recommend an approach that combines IPW with the commensurate prior (PS‐Commensurate) which we adopt in our study and explain in more detail below.

First, given a model for the propensity score, we can derive weights, $w_{i}$, for the control group subjects as follows:

$$w_{i}={\tilde{S}}_{i}+\frac{e_{i}\left(1-{\tilde{S}}_{i}\right)}{1-e_{i}}$$

Evidently, all subjects participating in the current trial get assigned a weight of 1 while the external controls are assigned weights $w_{i}=e_{i}/\left(1-e_{i}\right)$. This has two consequences: (1) the external control group subjects are reweighted based on the individual similarities in terms of baseline covariates included in the propensity score model; (2) additionally, the total nominal sum of weights for the external control group is scaled to equal the internal control sample size in expectation, thus limiting the influence of the external data on control group parameter estimation. In the next step, we apply the commensurate prior to the PS‐weighted sample. In the following, let $\theta ≔E\left[Y^{\left(0\right)}|S=0\right]$ be our parameter of interest that is the expected potential outcome under the control treatment in the current trial. The commensurate prior for this parameter, using the weighted data, is given as

$$p\left(\theta |D_{1},\theta_{1},\tau \right)\propto p_{w}\left(D_{1}|\theta_{1}\right)p\left(\theta |\theta_{1},\tau \right)p_{0}\left(\theta \right)$$

Here, $p_{w}\left(D_{1}|\theta_{1}\right)$ denotes the PS‐weighted version of the historical data likelihood, that is $p_{w}\left(D_{1}|\theta_{1}\right)=\prod_{i\in D_{1}}p{\left(y_{i}|\theta_{1}\right)}^{w_{i}}$. In practice, we need to plug in the weights derived from an estimated propensity score model ${\hat{w}}_{i}$. The initial prior, $p_{0}\left(\theta \right)$, is typically chosen to be uninformative. The crucial component in this formulation, however, is $p\left(\theta |\theta_{1},\tau \right)$. It induces a prior distribution for θ that is centered around the matching parameter from the historical data, $\theta_{1}$. The amount of borrowing information from the historical data is then governed by the *commensurability parameter*, τ, which translates to the precision (inverse variance) of that distribution. Therefore, as τ→0, the impact of the historical data diminishes. Conversely, as τ→∞, θ converges to $\theta_{1}$, resulting in pooling of the current and historical data. To allow the data to guide the quantification of commensurability an additional hyperprior, p(τ), gets added to the model specification. The joint prior distribution for θ and τ is then given by

$$p\left(\theta ,\tau |D_{1},\theta_{1}\right)\propto p\left(\theta |D_{1},\theta_{1},\tau \right)p\left(\tau \right)$$

Typically, a normal distribution is chosen for $p\left(\theta |D_{1},\theta_{1},\tau \right)$. For the hyperprior, p(τ), different options that respect the support of the parameter are possible. Here, we opt for a Gamma prior specification, that is, τ∼Gamma(0.001, 0.001), aligning with common practices in the literature [22]. Depending on the type of outcome and corresponding distributional assumptions, prior specifications for additional parameters may be required. The posterior kernel of θ is finally obtained by multiplying this joint prior distribution with the current data likelihood $p\left(D_{0}|\theta \right)$. To ultimately make inference on the treatment effect of interest, Δ, we can integrate this prior specification into a regression model. There, θ is represented by the intercept, $\beta_{0}$, and Δ corresponds to the coefficient $\beta_{a}$ associated with the treatment variable. We can then use a suitable Markov chain Monte Carlo (MCMC) algorithm to obtain posterior samples of all parameters of interest.

The integration of propensity scores into Bayesian models has been criticized from a conceptual point of view [23, 24]. A genuine Bayesian model requires a joint posterior distribution of all model parameters, that is the parameters of the outcome model as well as those of the propensity score model. Due to the two‐stage procedure, however, this is not the case here and there is not definite solution to this problem yet [23]. Nonetheless, as Li et al. argue, it is OK for a method to be only “quasi‐Bayesian”, when the benefits are clear compared to fully Bayesian solutions or other alternatives. Moreover, it is worth mentioning that the commensurate prior model has been developed for the purpose of borrowing information from a single external source only [19]. It has been extended to accommodate information borrowing from multiple sources under the assumption that these different sources are homogeneous [25] though recent work has aimed to address this limitation [26].

### 3.2 Propensity Score Weighted Multi‐Source Exchangeability Models

The PS‐weighted multi‐source exchangeability modeling (PS‐MEM) framework [12] has some similarities with the PS‐Commensurate prior introduced in the previous section. It also exhibits a two‐stage procedure and, in fact, applies the same initial PS adjustment step. The PS‐MEM approach can be distinguished from PS‐Commensurate, however, in that it employs a form of Bayesian model averaging (BMA) in the second stage to borrow historical data. This idea has first been suggested by Kaizer et al. [27] from whom it received the label *multi‐source exchangeability modeling* (MEM). To explain the concept of the MEM approach, we introduce additional notation, following Wei et al. First, let $\theta_{s}≔E\left[Y^{\left(0\right)}|S=s\right],s=0,1,\ldots ,S^{\prime }$ denote the expected potential outcome under the control treatment in the s'th study. The initial step within this framework is to list all possible exchangeability configurations which are denoted by $M_{k},k=1,\ldots ,K$. Exchangeability, in this context, is defined as equality of the respective mean parameters. Within each exchangeability configuration, there are $L_{k}$ clusters, which are sets of control groups sharing a common mean parameter $\theta_{l_{k}},l_{k}=1,\ldots ,L_{k}$. We illustrate the above by adopting the example of two historical trials by Wei et al. In this scenario, there are K=5 possible exchangeability configurations:

$$\begin{array}{ll} M_{1}: & \;\theta_{0}=\theta_{1}=\theta_{2}\\ M_{2}: & \;\theta_{0}=\theta_{2}\neq \theta_{1}\\ M_{3}: & \;\theta_{0}=\theta_{1}\neq \theta_{2}\\ M_{4}: & \;\theta_{0}\neq \theta_{1}=\theta_{2}\\ M_{5}: & \;\theta_{0}\neq \theta_{1}\neq \theta_{2} \end{array}$$

We can then have $L_{1}=1$ up to $L_{5}=3$ clusters within one configuration in the two most extreme scenarios. These correspond to situations where all data sets get pooled ($M_{1}$) or are treated separately ($M_{5}$). The idea of the MEM framework is not to decide a priori for a single configuration but to fit all of these models and assess their plausibility via BMA. To apply this idea, we require the joint weighted likelihood of the control group data under model $M_{k}$ which is given by the product of the different clusters' likelihoods under that configuration:

$$p_{w}\left(y|\theta_{k},M_{k}\right)=\prod_{l_{k}=1}^{L_{k}}p_{w}\left(y_{l_{k}}|\theta_{l_{k}},M_{k}\right)$$

The weighted cluster likelihood, in turn, is obtained by the likelihoods of the individual data sources that belong to the respective cluster $l_{k}$ under this model:

$$p_{w}\left(y_{l_{k}}|\theta_{l_{k}}\right)=\prod_{s\in l_{k}}p_{w}\left(y_{s}|\theta_{l_{k}}\right)$$

Finally, the likelihood of one data source belonging to a cluster is given by the corresponding individual contributions, also impacted by their PS weights $w_{i}$:

$$p_{w}\left(y_{s}|\theta_{l_{k}}\right)=\prod_{i\in D_{s}}p{\left(y_{i}|\theta_{l_{k}}\right)}^{w_{i}}$$

In order to apply BMA and obtain posterior model probabilities, we need to compute the marginal likelihoods (also called evidence) for each model, which is given by the product of the clusters' marginal likelihoods:

$$p_{w}\left(y|M_{k}\right)=\prod_{l_{k}=1}^{L_{k}}p_{w}\left(y_{l_{k}}|M_{k}\right)$$

These can, in turn, be obtained by integrating the respective likelihoods over the prior distribution of $\theta_{l_{k}}$:

$$p_{w}\left(y_{l_{k}}|M_{k}\right)=\int_{\Theta }p_{w}\left(y_{l_{k}}|\theta_{l_{k}}\right)p\left(\theta_{l_{k}}\right)d\theta_{l_{k}}$$

Given these ingredients as well as prior model probabilities, $p\left(M_{k}\right)$, the posterior model probabilities can be computed as:

$$p_{w}\left(M_{k}|y\right)=\frac{p_{w}\left(y|M_{k}\right)p\left(M_{k}\right)}{\sum_{k^{\prime }=1}^{K}p_{w}\left(y|M_{k^{\prime }}\right)p\left(M_{k^{\prime }}\right)}$$

For binary, Poisson, and Gaussian data, Wei et al. provide analytical expressions for computing the marginal likelihood (see Table 1 therein). However, for Gaussian data, this requires a practical approximation. It is assumed that the variance of the Gaussian likelihood model is known, but it needs to be estimated from the data in practice. The advantage of this procedure is that it avoids the need for expensive MCMC sampling and that a closed‐form solution for the marginal likelihood can be derived. From the posterior model probabilities, we can then derive the posterior exchangeability probability for the individual studies:

$$\alpha_{s}≔p_{w}\left(\theta_{s}=\theta_{0}\right)=\sum_{k=1}^{K}p_{w}\left(M_{k}|y\right)I\left(\theta_{s}=\theta_{0}|M_{k}\right)$$

**TABLE 1:** Comparative characterization of the three borrowing methods.

	PS‐Commensurate	PS‐MEM	BART
Borrowing Mechanism	Informative prior distribution	Parametric model uncertainty (BMA)	Nonparametric model uncertainty
Use of baseline covariates	PS weighting	PS weighting	Nonparametric covariate adjustment
Multiple historical datasets?*	×	✓	✓
Interpretability	✓	✓✓	×
Computation	MCMC	Analytical	MCMC
Outcome types	Broadly applicable	Limited (Gaussian, binary, Poisson)	Broadly applicable

* In terms of selective borrowing.

The posterior distribution for our parameter of interest, $\theta_{0}$, can be obtained by integrating the posterior exchangeability probabilities into the likelihood functions for $\theta_{0}$ and multiplying it with an initial prior $p\left(\theta_{0}\right)$: 


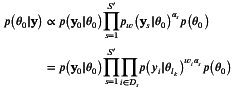




These expressions provide two interesting insights. First, the historical data likelihoods being raised to the power of their posterior exchangeability probabilities reveals some similarities between MEM and the power prior [2]. Second, we can discern how the borrowing takes place at the subject level ($w_{i}$) through the PS adjustment as well as at the level of the historical data set as a whole ($\alpha_{s}$) based on the outcome similarity. Furthermore, a useful quantity that can be calculated from this posterior is the *effective historical sample size* (EHSS):

$$\text{EHSS}=\sum_{s=1}^{S^{\prime }}\sum_{i\in D_{s}}\alpha_{s}w_{i}$$

To finally conduct posterior inference for Δ, we obtain a separate posterior distribution for $E\left[Y^{\left(1\right)}\right]$ and combine the results. Compared to the PS‐Commensurate prior, the same conceptual critiques apply. An advantage of PS‐MEM is that it can incorporate multiple historical studies that can receive differential weights.

### 3.3 Bayesian Additive Regression Trees

Bayesian additive regression trees (BART) [28] is a method that merges approaches from Bayesian statistics and machine learning. After its introduction it has quickly emerged as a popular model for causal inference tasks [17, 29]. Unlike the two methods introduced before, however, it has not originally been developed for the intent of borrowing information from external data sources in an RCT. This application of BART was recently proposed by Zhou and Ji. Fundamentally, BART aims to approximate an unknown mean function via a non‐parametric sum‐of‐trees model:

$$Y=f\left(x\right)+\varepsilon \approx \sum_{j=1}^{m}g_{j}\left(x\right)+\varepsilon ,\;\varepsilon ∼N\left(0,\sigma^{2}\right)$$

Specifically, a single regression tree model is defined as $g_{j}\left(x\right)=g\left(x|T_{j},M_{j}\right)$, where T denotes a binary tree with interior decision rules and $M=\left\{\mu_{1},\mu_{2},\ldots ,\mu_{b}\right\}$ is a corresponding set of values belonging to the b terminal nodes of that tree. In a single‐tree model, these values would represent the estimated conditional expectations of the outcome variable of interest. However, in a sum‐of‐trees model, each tree acts as a weak learner, contributing only a portion to modeling the overall conditional mean E[Y|x]. A key component of BART, beyond representing f(x) as a sum‐of‐trees model, is the use of regularization priors to prevent overfitting. Specifically, a regularization prior is imposed on the trees, $T_{j}$, ensuring that each tree remains relatively shallow with few terminal nodes only. Similarly, the priors for the parameters in $M_{j}$ are specified to induce shrinkage. Chipman et al. have developed a tailored MCMC backfitting algorithm for this model to obtain posterior draws that can be used for inference afterwards. For a comprehensive description of both the prior specifications and the MCMC algorithm we refer readers to this reference. The BART model has two main attractive properties: First, its nonparametric nature allows it to automatically detect and model various types of covariate effects, including nonlinear and interaction effects. This makes it particularly useful when little is known about the functional relationships beforehand. Second, by leveraging the Bayesian framework and utilizing an MCMC algorithm, BART provides uncertainty estimates for all derived quantities of potential interest.

Zhou and Ji have suggested using BART for borrowing historical control data in RCT settings. To do so, they first specify separate BART models for the treatment and control group, respectively. The treatment group model, $f_{1}\left(x\right)$, models the conditional expectation, $E\left[Y^{\left(1\right)}|x\right]$ based on all relevant baseline characteristics. The model for the control group, $f_{0}\left(x,s\right)$, is set up in a similar manner but differs in some details. First, it is important to note that $f_{0}$ is estimated based on the pooled control group data from the current and historical clinical trials. For this reason, it also contains the study indicator, s, as explanatory variable in addition to the baseline covariates. The idea of this model formulation is the following: If the data from the current and historical trials are not exchangeable, the BART model can detect this and make a split based on the study indicator. On the other hand, if there are no signs of non‐exchangeability, no split should be made and the data sets are effectively pooled. Moreover, BART might be able to detect conditional exchangeability, that is, identify specific baseline covariate configurations for which exchangeability holds whereas this might not hold for the entire population. Once the two models have been fitted, posterior draws of $\Delta_{c}\left(x_{i}\right)$ and, more specifically, $\Delta_{c}\left(x_{i},s_{i}=0\right)$ are readily available. Posterior draws of Δ can then be obtained by standardizing the CATEs, $\Delta_{c}\left(x_{i},s_{i}=0\right)$, over the corresponding multivariate covariate distribution for which Zhou and Ji propose to use the Bayesian bootstrap [30]. In essence, this means that (posterior) weights are generated as $w^{*}∼\mathrm{Dir}\left(1_{n_{0}}\right)$ for each draw where $w^{{*}^{\prime }}1_{n_{0}}=1$ holds. Then, the posterior draws of Δ are calculated as $\Delta =\sum_{i=1}^{n_{0}}w_{i}^{*}\Delta_{c}\left(x_{i}\right)$. Like PS‐MEM, BART also has the capability to leverage more than one historical data source.

### 3.4 Summary

To summarize, we contrast the three methods across their core conceptual and methodological features. First, the borrowing mechanism of the different approaches differs. PS‐Commensurate employs the standard informative prior distribution framework, whereas PS‐MEM and BART frame the problem through the lens of model uncertainty, with the latter utilizing a non‐parametric approach. Regarding interpretability, PS‐Commensurate utilizes a commensurability parameter whose posterior distribution reflects the degree of borrowing, though its meaning remains context‐dependent. BART is generally more difficult to interpret in this context. In contrast, PS‐MEM provides the most intuitive metrics, specifically posterior exchangeability probabilities and effective historical sample size. Finally, the methods vary in implementation and scope. While PS‐Commensurate and BART rely on MCMC sampling, PS‐MEM offers analytical solutions. However, this efficiency restricts PS‐MEM to Gaussian, binary, and Poisson data. Conversely, PS‐Commensurate and BART are applicable to a broader range of outcome types, though BART may require specialized software packages. Table 1 provides an overview of these and further methodological features.

## 4 Simulation Study

After we have presented three candidate methods for borrowing historical control data in RCTs in the previous section, we now want to evaluate their performance and behavior in a simulation study. For this, we follow the ADEMP (aims, data‐generating mechanisms, estimands and other targets, methods, performance measures) structure introduced by Morris et al. [31].

### 4.1 Design

#### 4.1.1 Aims

The aim of this simulation study is to compare the performance of three different covariate‐adjusted BDB methods. On the one hand, the goal of these methods is to improve efficiency of treatment effect estimation and increase power under non‐null effect scenarios. On the other hand, dynamic borrowing involves the risk of biased effect estimation and inflated Type I error rates. In this regard, we are particularly interested in how the methods perform under different scenarios that can include observed and unobserved sources of confounding. Furthermore, we want to consider situations in which covariates are included in the dynamic borrowing procedure that do not have a relationship with the outcome of interest and determine its impact on performance. In summary, the following questions should be addressed:

- How efficiently do the different methods borrow historical information when appropriate?
- How robust are the methods when there are discrepancies between the current and historical data?
- How is the performance of the methods impacted when uninformative covariate information is used?

#### 4.1.2 Data‐Generating Mechanisms

To begin with, we consider the setting of an RCT with $n_{0}^{\mathrm{trt}}=60$ patients enrolled in the active treatment arm and $n_{0}^{\text{ctrl}}=30$ patients enrolled in the concurrent control arm. We assume that the outcome, y, is normally distributed with a common group‐wise marginal variance $\sigma_{y}^{2}=81$. Assuming different effect sizes for the treatment, Δ∈{0,3.5,4.5}, a one‐sided two‐sample *t*‐test with significance level α=2.5% would have a power of roughly 40% and 60% in the corresponding non‐null scenarios. By leveraging historical control group data from one historical RCT, the goal is to increase these powers. For this, we consider different historical data sample sizes, $n_{1}^{\text{ctrl}}\in \left\{30,60,120\right\}$. The outcome data are generated from the following linear model:^1^

$$\begin{array}{cc} y_{s} & =\beta_{0}+\beta_{1}x_{1,s}+\beta_{2}x_{2,s}+\beta_{3}x_{3,s}+\Delta \cdot a\\ & \;+u_{s}+\varepsilon ,\;\varepsilon ∼N\left(0,7^{2}\right),\;s\in \left\{0,1\right\} \end{array}$$

We set the intercept of the model and the regression coefficients referring to the baseline covariates as $\left(\beta_{0},\beta_{1},\beta_{2},\beta_{3}\right)=\left(2, 1, 1, 0\right)$. The covariates themselves, $\left(x_{1,s},x_{2,s},x_{3,s}\right)$, are generated from a multivariate normal distribution with the following parameters:

$$\mu_{s}=\left(\begin{array}{c} 8\\ 8+s\cdot \delta_{2}\\ 8+s\cdot \delta_{3} \end{array}\right),\;\sum =\left(\begin{array}{ccc} 6 & 2 & 0\\ 2 & 6 & 0\\ 0 & 0 & 6 \end{array}\right)$$

These specifications have the following implications. The covariates $x_{1}$ and $x_{2}$ are prognostic for the outcome y and have correlation $\rho_{1,2}=1/3$. The variance of the linear predictor term equals 16 such that $R^{2}\approx 0.2$, that is, for each treatment group roughly 20% of the variation in the outcome variable can be explained by baseline covariates. The mean of $x_{2}$ can differ in the historical data relative to the current trial by $\delta_{2}$, for which we consider values {0,±1,±2,±3}. Since $\beta_{2}=1$, these values directly correspond to the different degrees of observed confounding in terms of the mean difference. Similar considerations are made for the latent variable $u_{s}$ through which we induce unobserved confounding. It is generated as $u_{s}∼N\left(0+s\cdot \delta_{u},4^{2}\right)$ where $\delta_{u}$ can take values from the same set as $\delta_{2}$. Negative values of $\delta_{2}$ and $\delta_{u}$ then increase the Type I error risk because they make the concurrent control group appear more inferior than it actually is when pooling the data. On the other hand, positive values of $\delta_{2}$ and $\delta_{u}$ can deteriorate the analysis by decreasing the power of the test. In this regard, we do not consider a fully factorial design but only consider scenarios where the two sources of confounding are geared toward the same direction. Thus, we keep scenarios where $\delta_{2}\cdot \delta_{u}>=0$ holds. Lastly, we examine mean shifts, $\delta_{3}$, for the non‐prognostic covariate $x_{3}$ as well. Here, the idea is to inspect how the different methods react to covariate information that might differ between the respective sources but is actually uninformative for the outcome of interest. Since $x_{3}$ is uninformative we do not need to consider both positive and negative values for $\delta_{3}$. Hence, we only look at $\delta_{3}\in \left\{0,1,2,3\right\}$. When we combine all of these factors and subset these combinations as described above we end up with a total of 1116 scenarios to simulate (see Table 2).

**TABLE 2:** Factors and their values making up the different simulation scenarios.

Parameter	Description	Values	Number of values
$n_{1}^{\text{ctrl}}$	Historical control group sample size	30, 60, 120	3
Δ	Treatment effect size	0, 3.5, 4.5	3
$\delta_{2}$	Mean shift of $x_{2}$ (prognostic), causing observed confounding	{0,±1,±2,±3}	7
$\delta_{3}$	Mean shift of $x_{3}$ (non‐prognostic), not causing any confounding	0, 1, 2, 3	4
$\delta_{u}$	Mean shift of latent variable u, causing unobserved confounding	{0,±1,±2,±3}	7
Number of scenarios (fully factorial): 1764 Number of scenarios (selected subset): 1116			

#### 4.1.3 Estimands and Other Targets

Our estimand of interest is the average treatment effect in the current trial, Δ. As we consider a clinical trial setting where a binary decision regarding the treatment efficacy needs to be made we also target the hypotheses pair:

$$H_{0}:\Delta \leq 0\;\mathrm{vs}.\;H_{1}:\Delta >0$$

#### 4.1.4 Methods

The methods of our main interest that will be compared have all been introduced in Section 3, namely the PS‐weighted commensurate prior, PS‐weighted multi‐source exchangeability models, and Bayesian additive regression trees. In addition, we include two benchmark methods that represent the two most extreme ends of (not) using historical control data, that is, a current and pooled data analysis, respectively. For these, we employ simple linear models that only include the treatment covariate.

#### 4.1.5 Performance Measures

The ultimate goal of dynamic borrowing is to increase the power of hypothesis tests of treatment effect estimates while the biggest concern is the potential of Type I error inflations. Therefore, our main performance measures are the Type I error rate and power. Performance measures concerning point estimators of the ATE, $\hat{\Delta }$, such as bias, empirical standard error, and mean squared error, are also considered for additional insights.

### 4.2 Computational Details

For each of the scenarios described in Section 4.1.2, we generate $N_{\mathrm{sim}}=2000$ data sets. Assuming a point estimate of the Type I error rate at the exact nominal level of 2.5%, this gives the corresponding 95% binomial confidence interval [1.8%,3.2%]. In other areas of methodological research, this level of precision might be considered insufficient. However, in the context of dynamic borrowing methods, we expect potential Type I error inflations to be relatively severe and therefore argue that this number of simulation repetitions is adequate, especially when taking into account the large computational run time needed for this study.

The different methods outlined in Section 3 need to be equipped with additional prior specifications. We assume vague normal priors (N(0,σ=100)) for the historical data intercept and the treatment effect in the PS‐Commensurate model. For the corresponding residual standard deviation, we use a half‐Cauchy prior (${\text{Cauchy}}_{+}\left(\sigma =5\right)$). Regarding PS‐MEM, we assume equal prior model probabilities, which in our simulation settings corresponds to prior probabilities of 0.5. As for BART, we apply the hyperprior and hyperparameter specifications recommended by Chipman et al.

While for PS‐MEM all computations can be carried out analytically, we rely on MCMC sampling for the other two methods. For these, we run four chains with a warmup period consisting of 1000 draws and a posterior sampling period consisting of 2000 draws. All computations have been carried out in a high‐performance computing environment using the R programming language in version 4.5.0 [32]. To implement the PS‐Commensurate model we used the psborrow2 package [33] which uses the Stan probabilistic programming language [34] as backend for MCMC sampling. We implemented the PS‐MEM method by ourselves, aided by R scripts that the authors of this method provided along with their manuscript [12]. Lastly, we used the dbarts package [35] to implement the suggested BART approach by Zhou and Ji. Further direct and indirect dependencies that were required to implement the methods and run the simulation study have been tracked and managed via the renv package [36]. The code for this study is publicly available on GitHub (https://github.com/DavidJesse21/borrow‐causal).

#### 4.2.1 Results

Capturing all results of the simulation study in a single table or figure is infeasible due to the high‐dimensionality of the study design. Therefore, we analyze the results of the study incrementally and accordingly shift the focus toward the different aspects of interest. The results for the pooled data analysis are excluded as including them prohibited visual clarity and a proper comparison of the methods of main interest.

We start by focusing on the effect of the degree of unobserved confounding ($\delta_{u}$) on the Type I error and power of the different methods while also taking into account the historical data sample size ($n_{1}^{\text{ctrl}}$). These results are shown in Figure 1 where each data point represents one scenario per method. Unsurprisingly, no systematic variations can be observed for the current data analysis as it does not utilize the historical data. In case of no unobserved heterogeneity at all, all methods are able to control the Type I error in most scenarios with some exceptions regarding PS‐Commensurate and PS‐MEM. As $\delta_{u}$ increases toward the negative direction, that is, making the control group seem more inferior than it is, we can observe Type I error inflations for some of the borrowing methods. Averaged over all respective scenarios, the Type I error of PS‐Commensurate increases from 2.9% ($\delta_{u}=0$) to 4.1% ($\delta_{u}=-1$), 5.1% ($\delta_{u}=-2$), and 6.2% ($\delta_{u}=-3$), respectively. For PS‐MEM, these inflations are less pronounced reaching an average of 3.7% ($\delta_{u}=-3$) only. Interestingly, there are no or only minor increases in the Type I error for BART. In the opposite direction, we see that the actual Type I error can fall below the nominal level of 2.5%, in particular for PS‐Commensurate. Likewise, the power is affected in non‐null scenarios by varying degrees of unobserved confounding, again most pronounced for PS‐Commensurate. One notable observation in this regard is that for small positive values of $\delta_{u}$ ($\delta_{u}=1$) including the historical data still results in some power gains compared to the current data analysis. Thus, in these scenarios the precision gain by incorporating the historical data is larger than the induced bias resulting in an underestimation of the true treatment effect. For the remainder of the comparison of power of the different methods, we focus on scenarios with no unobserved confounding ($\delta_{u}=0$) where information borrowing should be most pronounced. As we would expect, in these scenarios all methods have an increased power compared to the current trial data analysis. Across all scenarios considered in this respect, the mean power gains are 5.3% for PS‐MEM, 9.3% for PS‐Commensurate, and 7.7% for BART. When further stratifying the scenarios by the historical data sample size, the mean power gains for PS‐MEM are 5.1% ($n_{1}^{\text{ctrl}}=30$), 5.3% ($n_{1}^{\text{ctrl}}=60$), and 5.6% ($n_{1}^{\text{ctrl}}=120$). On the other hand, for PS‐Commensurate these gains are 8.4%, 9.3%, and 10.3%, respectively. Hence, the power gains from borrowing historical control data do not notably increase for PS‐MEM with growing historical data sample size. For PS‐commensurate, while still small, we can observes such increases. Two factors account for this: first, propensity score weighting scales the nominal total weight of the historical controls to match the internal control group sample size in expectation; second, the respective Bayesian modeling approach modulates how much information then still gets incorporated. As for BART, power gains due to increasing historical data sample size are essentially non‐existing. Here, we have values of 7.9% ($n_{1}^{\text{ctrl}}=30$), 7.6% ($n_{1}^{\text{ctrl}}=60$), and 7.6% ($n_{1}^{\text{ctrl}}=120$). Because of this observation, we hypothesize that the observed power gains for BART can mainly be attributed to its capability to adjust for prognostic covariate effects. However, it appears to borrow only little information from the external data source, which would also explain the minor Type I error inflations in scenarios with inter‐study heterogeneity.

**FIGURE 1:**
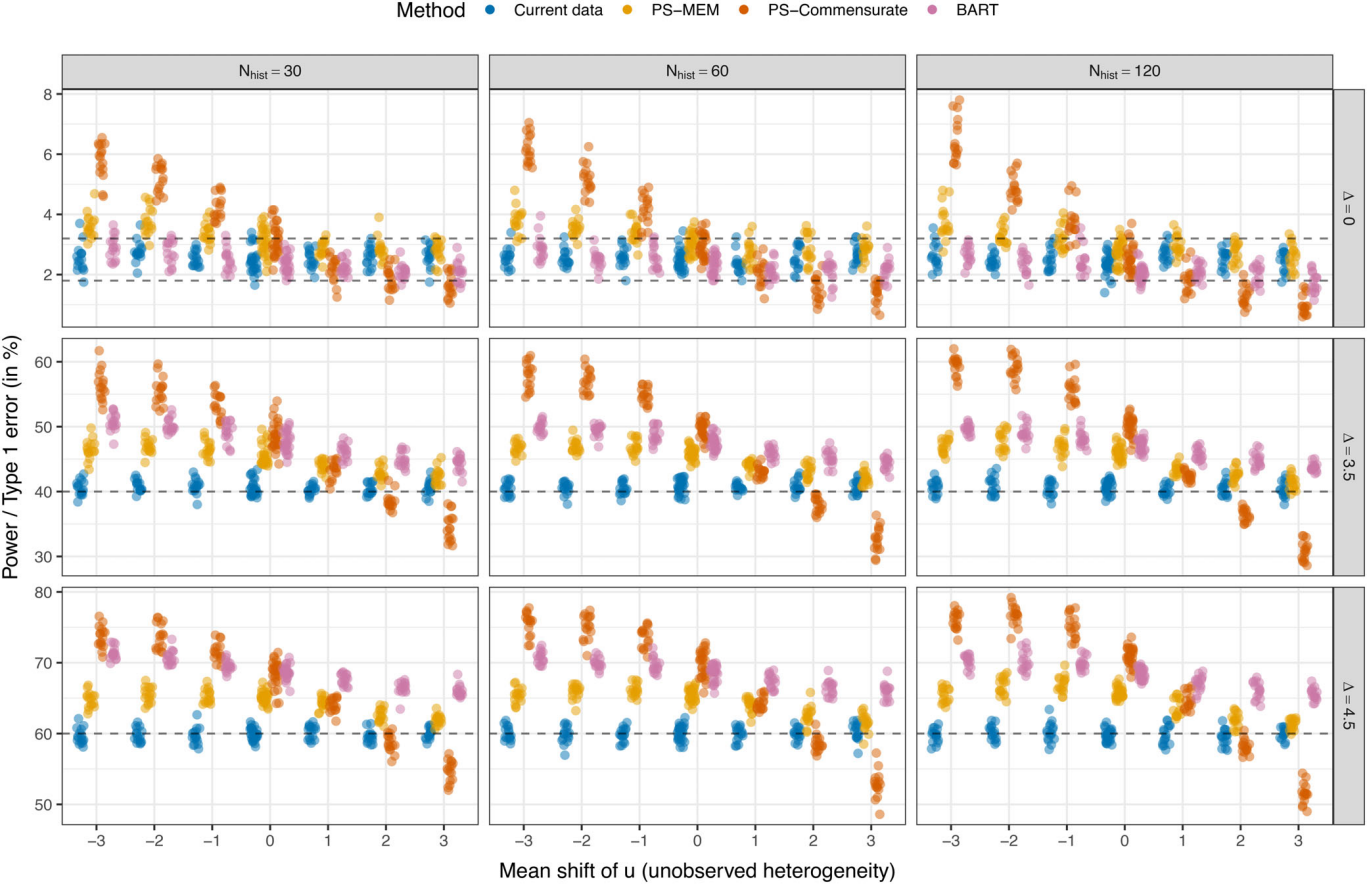
Rejection rates of different methods in % (nominal level α=2.5%) by historical data sample size, effect size, and degree of unobserved heterogeneity. The dashed lines depict the power of a two‐sample *t*‐test in cases of non‐null effect sizes and the 95% binomial confidence interval [1.5%,3.5%] around the nominal level in case of the null scenario.

We shift the focus toward the impact of observed confounding, that is, (mean) differences in the outcome distributions between the concurrent and historical control subjects that can be explained by the prognostic baseline covariate $x_{2}$. We provide a similar graphical presentation of the results in Figure 2 with the different values for $\delta_{2}$ on the x‐axis instead. First of all, it can be noted that, compared to Figure 1, there is a lot more variability within the different x‐axis strata w.r.t. the Type I error and power values and, conversely, less variability between the x‐axis strata. This indicates that the impact of observed confounding caused by prognostic baseline covariate is less severe and can be better handled than the unobserved confounding. Figure 3 provides further confirmation for this. There, the impact of $\delta_{u}$ and $\delta_{2}$ on the Type I error is compared for all methods by plotting the results for only those scenarios in which the respective other parameter is set to 0. Whereas there is a notable effect of $\delta_{u}$ on the Type I error, particularly for PS‐Commensurate, there is no obvious corresponding effect of $\delta_{2}$. Nonetheless, $\delta_{2}$ can have a notable impact on power, as shown in Figure 2. This effect is most obvious for PS‐Commensurate, where power decreases with larger absolute values of $\delta_{2}$. The power loss is driven by propensity score weight variability, thereby reducing statistical efficiency and inflating the variance of the control baseline estimate.

**FIGURE 2:**
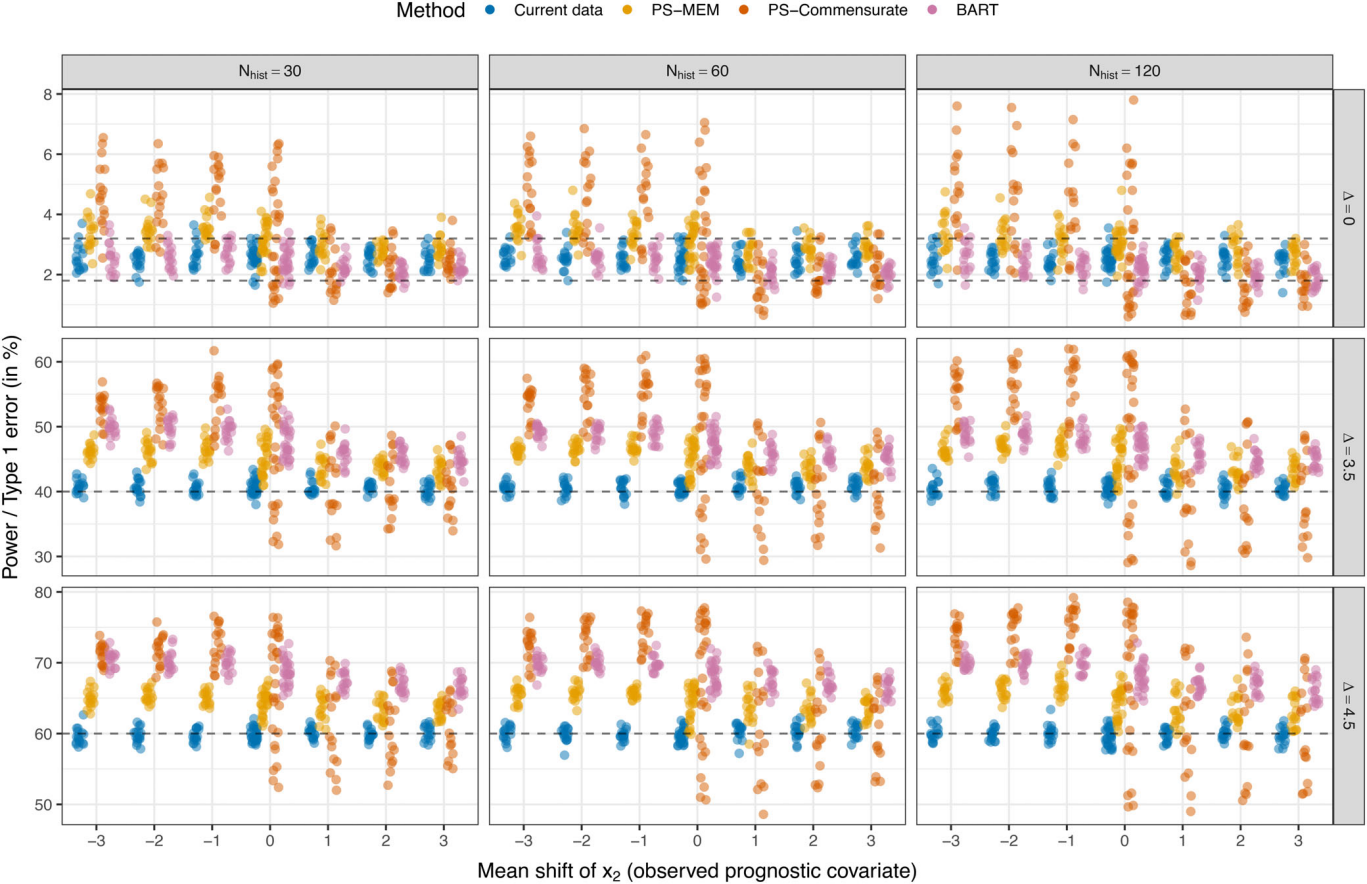
Rejection rates of different methods in % (nominal level α=2.5%) by historical data sample size, effect size, and degree of observed confounding. The dashed lines depict the power of a two‐sample *t*‐test in cases of non‐null effect sizes and the 95% binomial confidence interval [1.5%,3.5%] around the nominal level in case of the null scenario.

**FIGURE 3:**
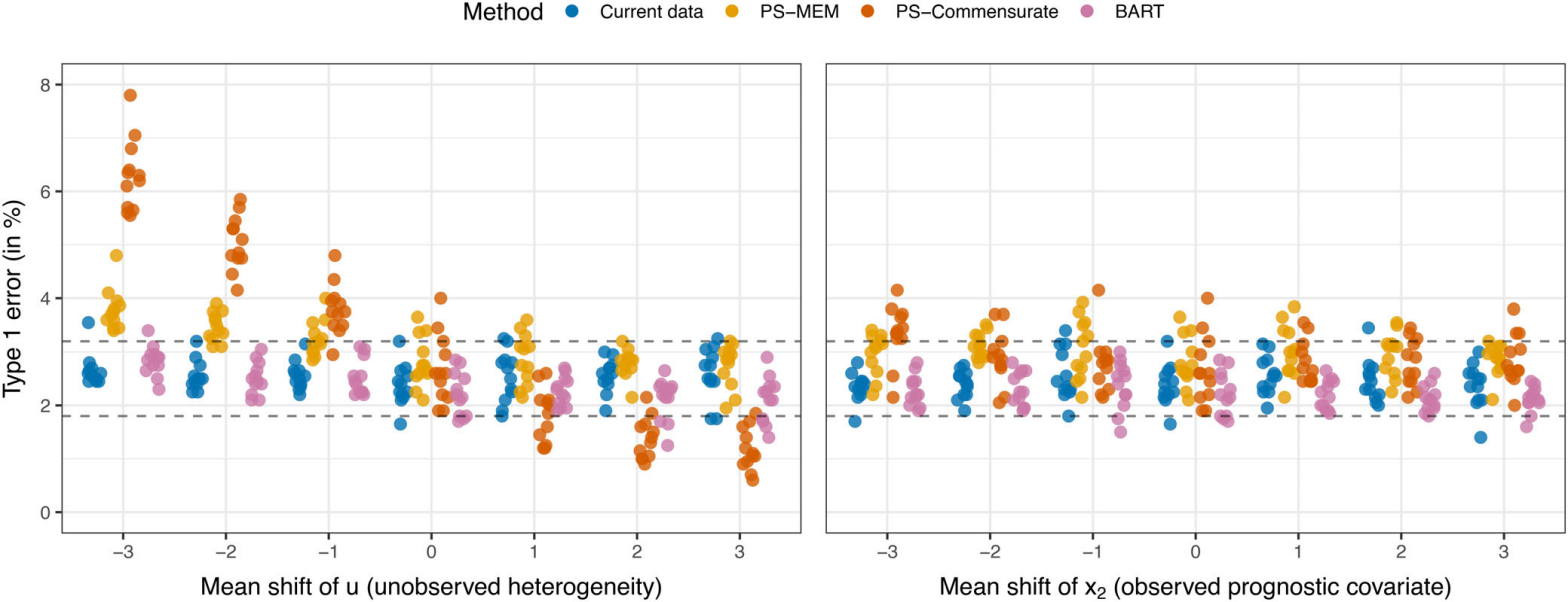
Comparison of the impact of observed and unobserved confounding on Type I error rates (nominal level α=2.5%) of the different methods when the respective other parameter is set to 0. The dashed lines depict the 95% binomial confidence interval [1.5%,3.5%] around the nominal level.

Next, we analyze the impact of $\delta_{3}$, that is, differences in the distribution of a baseline covariate between current and historical controls, that is not prognostic for the outcome. For this analysis, we again consider scenarios, in which other nuisance factors are eliminated, precisely scenarios for which $\delta_{u}=0$ and $\delta_{2}=0$ hold. We show corresponding results for performance metrics regarding the point estimation (bias, empirical standard error, and mean squared error [MSE]) in Figure 4. With increasing values of $\delta_{3}$, we can observe increases in the MSE for all methods, most notably for PS‐Commensurate, however. It can further be seen that these increases in MSE can be attributed to an increase in the (empirical) standard error whereas the bias seems to remain largely unaffected by this parameter. Hence, including a non‐prognostic baseline covariate does not introduce bias, but it reduces statistical precision. For weighting‐based approaches, it inflates weight variance and risks (near) positivity violations; for BART, it may increase model complexity and variance by splitting on irrelevant noise variables.

**FIGURE 4:**
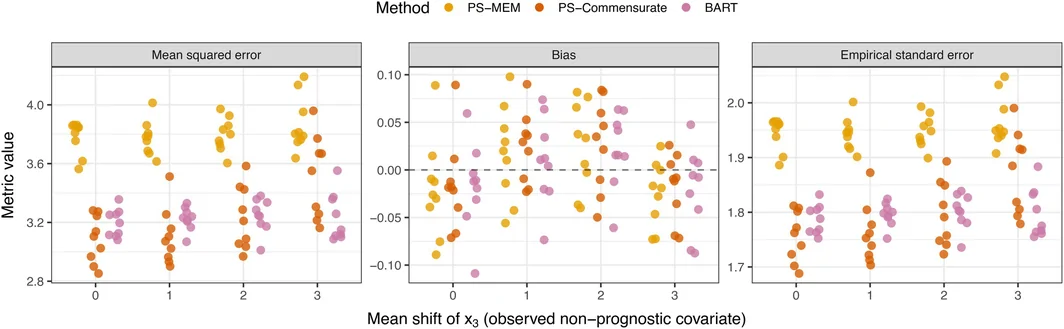
Impact of variation of observed covariate that is non‐prognostic on point estimation of the different methods. The plotted data only include scenarios in which $\delta_{u}=0$ and $\delta_{2}=0$ hold.

Based on the insights from the simulation results so far, we further analyze the effect of the historical data sample size to better understand if and how much the different methods actually borrow information from the historical control source. Under ideal conditions, a larger historical sample size is expected to improve parameter estimation and increase precision (e.g., as measured by MSE). Therefore, Figure 5 displays the MSE of the different methods against the historical data sample size for scenarios in which $\delta_{u}=0$ holds. For $n_{1}^{\text{ctrl}}=30$, we can see that BART generally has the lowest MSE of all methods, which, as mentioned before, can be attributed to its ability to adjust for covariates. However, there is no striking trend of a decreasing MSE for this method when $n_{1}^{\text{ctrl}}$ increases. This supports our hypothesis that, at least in our simulations, BART barely leverages any of the historical information it gets provided. When comparing these insights with the results from the simulation study conducted by the authors of this method [13] we can conclude that they are partially in agreement. Hence, in scenarios 4 and 5 that Zhou and Ji investigate the BART borrowing model does not have a larger power compared to the BART model that does not use any historical data. In the other scenarios (1–3), however, such power gains can be observed. One distinguishing aspect between these two groups of scenarios is the shape of the response surface. For the first group of scenarios (1–3) the response surfaces are more complex, including nonlinear and covariate‐by‐treatment interaction effects. The other group of scenarios have simpler relationships that is, linear (Scenario 4) or even flat (Scenario 5) response surfaces. To provide further evidence regarding these observations, we conducted two additional simulation studies in which the BART borrowing method is compared to a BART model using the current trial data only. A full description of these simulations and corresponding presentations of the results are provided in the supplementary material (Section S1). In both studies, we considered scenarios with perfect exchangeability between the concurrent and historical controls. The first study uses the same data‐generating mechanism as for the main study described in Section 4.1.2. For the second study, we replaced the linear predictor $\beta_{2}x_{2}$ with an exponential decay function, $b_{0}\exp\left(-b_{1}x_{2}\right)$, to include a scenario with nonlinearity. The results of both studies largely corroborate our conjecture about BART's information borrowing behavior. Thus, compared to a BART model that solely utilizes the concurrent control group data the power gains of the BART borrowing method range from 0.55% to 1.20%. Moreover, there is no discernible relationship between the historical control group sample size and the power gains for BART. Therefore, explaining when and why BART would (not) borrow historical information appears to be difficult. For PS‐MEM, the picture is similar to some extent. In cases of $n_{1}^{\text{ctrl}}=30$, its MSE is only slightly below that of the current data analysis. The corresponding decrease with growing historical data sample size for this method is rather marginal. On the other hand, for PS‐Commensurate the borrowing behavior is more recognizable. In particular, its MSE decreases with growing historical data sample size and gets to a similar level as BART at $n_{1}^{\text{ctrl}}=120$. From this we conclude that out of the three methods considered the borrowing mechanism of PS‐Commensurate works most effectively.

**FIGURE 5:**
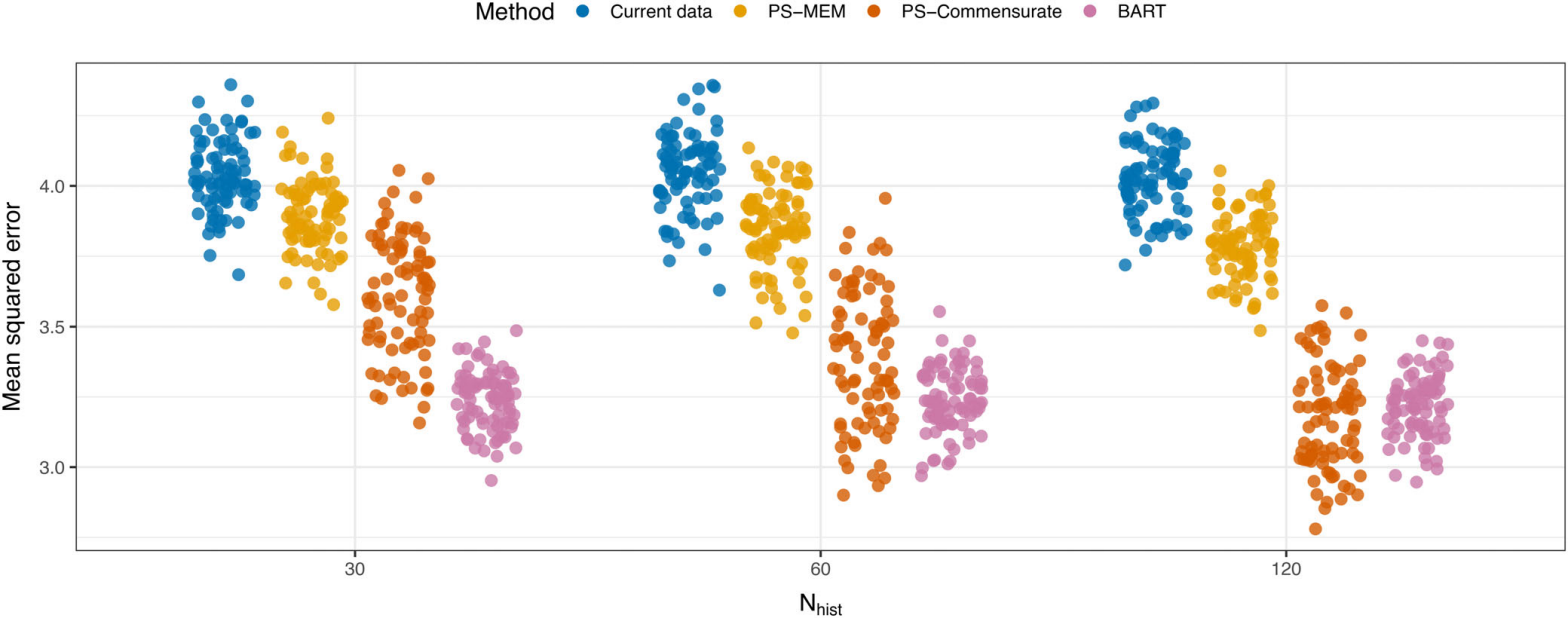
Impact of historical data sample size on mean squared error of the different methods. The plotted data only include scenarios in which $\delta_{u}=0$ holds.

A final observation we want to share concerns method failures, that is, situations in which a method is not able to produce the usual output, usually due to numerical issues. While there were no problems with PS‐Commensurate, BART, and the two benchmark methods, we detected 619 scenarios in which, for at least one iteration, PS‐MEM failed. We discuss the likely causes of this based on our graphical analysis in Figure 6. There, the relative frequency of method failures for PS‐MEM is plotted against $|\delta_{2}|+|\delta_{3}|$ and further stratified by $n_{1}^{\text{ctrl}}$. From this we can conclude that large discrepancies between the covariate distributions of the current and historical control group in combination with a small (historical control) sample size increase the likelihood of PS‐MEM to fail. The reason for this is that a smaller overlap in covariate distributions can cause extreme PS weights, even more so when the variability of the underlying model is large due to a small sample size. These extreme weights then affect the calculation of the weighted marginal likelihood used for BMA, which can lead to numerically infinite values in some cases. However, extreme PS weights are a known issue in the causal inference literature [37] and therefore suitable solutions for addressing this problem exist.

**FIGURE 6:**
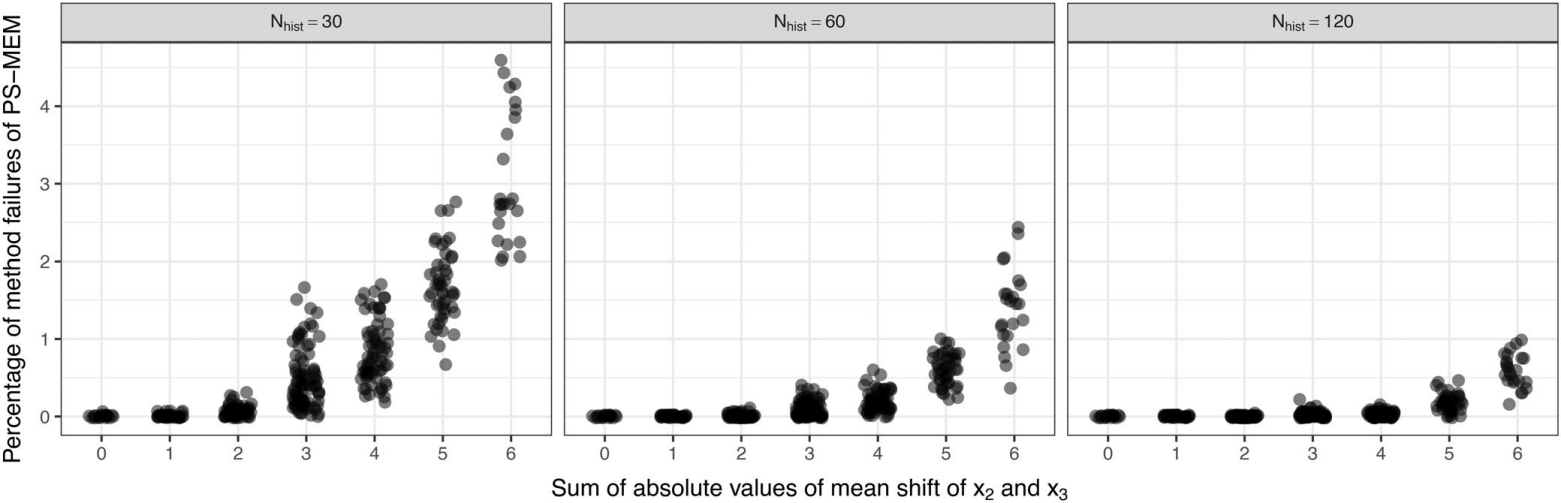
Percentage of method failures of PS‐MEM by mean covariate shifts ($|\delta_{2}|+|\delta_{3}|$) and historical data sample size.

## 5 Real Data Applications

While simulation studies are indispensable tools for methodological research, they are often criticized for not reflecting characteristics of real‐world data sets closely enough, which can potentially limit the transferability of the drawn conclusions to real scenarios [38]. For this reason, we complement the results from our simulation study with two illustrative applications of the methods to real clinical trial data. It needs to be noted that we have used anonymized versions of the actual clinical trial datasets, in which specific observations may have been removed or modified due to a high risk of being able to identify a patient. Thus, the results shown here may differ from previously published clinical results and no medical conclusions should be drawn from this paper. In both examples, we compare the three covariate‐adjusted BDB methods investigated in this paper to analyses of the current and pooled data sets using an unadjusted linear model to estimate the treatment effect. In addition to that, we also include the commensurate prior and MEM without PS weighting into the analysis as well as two BART models that use the current trial and pooled data sets, respectively. By doing this we aim to better understand and contextualize the results from the simulation study.

### 5.1 Rheumatoid Arthritis Trial

In the first example, we analyze anonymized data from the Phase 3 *OPTION* study (ClinicalTrials.gov identifier: NCT00106548) in which two dosages of tocilizumab (8 mg/kg and 4 mg/kg) were compared against placebo, all in combination with methotrexate (MTX), for treating patients with moderate to severe active rheumatoid arthritis. While in the original study ACR20 (American College of Rheumatology 20) response after 24 weeks has been the primary endpoint, we consider the change in the disease activity score (DAS) at Week 12 compared to baseline as the outcome of interest in this example. Moreover, we only compare the two tocilizumab groups (8 mg/kg vs. 4 mg/kg) and do not take the actual placebo control arm into account. The 8 mg group is then considered the active treatment arm while the 4 mg group acts as the control group in this example. The targeted estimand is the corresponding mean difference between these two groups. After considering those patients who had DAS measurements at both, Week 12 and baseline, the data set contains 99 subjects in the 8 mg/kg group and 94 subjects in the 4 mg/kg group. The goal of this illustrative example is to further understand some behaviors of the methods that we noticed in the simulation study and did not anticipate before. This refers particularly to situations of perfect exchangeability between the current and historical data sources in which much information borrowing would be expected. Therefore, we emulate a scenario in which there is a concurrent and a historical control group that should, in principle, have similar characteristics and therefore make it possible to leverage the external information to gain efficiency. To do this, we split the 4 mg/kg arm into two roughly equally sized groups based on the date the patients entered the trial. Patients who entered the trial before or at the median start date within that group are assumed to come from a previous clinical trial while the rest of the patients remain part of the concurrent control arm. As a result, we end up with 48 concurrent and 46 “historical” control group subjects. The set of baseline covariates used in this example contains the DAS baseline measurement, the duration of the disease, smoking status (yes/no), and a rheumatoid factor test result (positive/negative).

The results obtained by applying the different methods to this data set are presented in Figure 7. First and foremost, all methods come to the same conclusion of no treatment benefit when comparing the 8 mg/kg dose to the 4 mg/kg one, which is not surprising when looking at the results of the “pooled” analysis. The commensurate priors (PS‐weighted and unweighted) leverage some of the historical information, leading to point estimates (posterior means) that are roughly the average of the current and pooled data estimates. Likewise, the width of the credible intervals is in between those of the current and pooled data analyses. The PS weighting has a minor impact on the results, shifting the estimate slightly toward the current data estimate, but it is hard to judge whether this difference is systematic. The MEM approaches borrow considerably more historical information in this example than they did in the simulation study. For the PS‐weighted version, we obtain a posterior exchangeability probability of 52% while for the unweighted version it is 49%. For both methods, we have an effective historical sample size of 24 patients (rounded). Based on these results and further experiments (Section S.3 in the supplementary material) we conclude that the discrepancies in the amount of borrowing are related to the scale of the outcome (i.e., the unit of measurement). Thus, the smaller the scale the more historical information PS‐MEM tends to borrow. This is because PS‐MEM always uses flat priors for the mean parameters for Gaussian data. Since marginal likelihoods and, as a consequence, posterior model probabilities are sensitive to prior specifications (see e.g., [39]), changing the scale of the outcome without changing the priors of the parameters leads to different results. A practical strategy to mitigate this problem while retaining the original PS‐MEM procedure could be to standardize the outcome data before applying this method. Another noticeable observation regarding MEM and PS‐MEM is that the credible intervals are as narrow as the confidence interval of the pooled data model. While MEM and PS‐MEM borrow more historical information in this example it also seems like the uncertainty gets underestimated as they still use less information than the pooled model to construct those credible intervals. Regarding BART, our findings from the simulation study seem to be confirmed. Thus, like for the other dynamic borrowing methods we can observe a shifted treatment effect estimate and shorter credible intervals compared to the unadjusted current data analysis. However, there is virtually no difference compared to the results of the current trial BART model. Considering the results of the pooled BART model we get an impression of how the results could differ if the BART borrowing model would actually leverage some of the historical information in this example. This underpins our findings from the simulation study that efficiency gains can be made by using BART due to covariate adjustment but no or only little historical information is borrowed to achieve the same goal.

**FIGURE 7:**
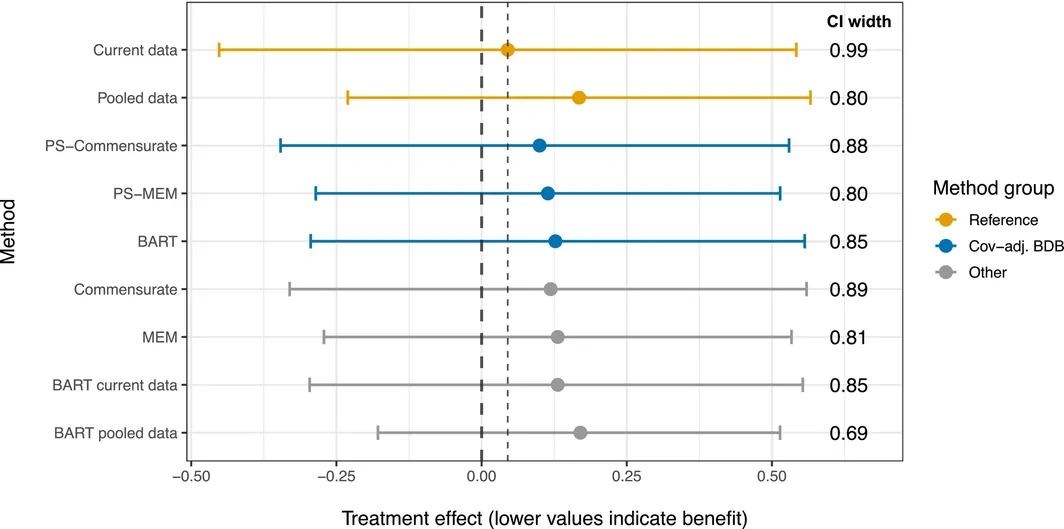
Point estimates and 95%‐confidence/credible intervals of different methods applied to the anonymized rheumatoid arthritis data. The thick dashed line references the null effect and the thin dashed line references the estimated effect using the current trial data only.

### 5.2 Systemic Sclerosis Trial

The second example comes closer to an actual scenario in which dynamic borrowing methods could be considered a serious option. Here, we analyze anonymized data from two clinical trials in systemic sclerosis, a rare autoimmune disease [40]. The data come from the two Phase 3 trials *FaSScinate* (ClinicalTrials.gov identifier: NCT01532869) and *FocuSSced* (ClinicalTrials.gov identifier: NCT02453256) that investigated the effect of tocilizumab in adult patients with systemic sclerosis compared to placebo. In both trials, the participants were randomized to a tocilizumab or a placebo group. For this example, we treat the FocuSSced trial as our current trial of interest and aim to supplement it with historical control subjects from the FaSScinate study which started 3.5 years before. The sample sizes in this scenario are 104 patients in the tocilizumab group, 106 patients in the concurrent placebo arm, and further 44 patients that were assigned to placebo in the FaSScinate study. Like in the FocuSSced trial, we consider the change from baseline in the modified Rodnan skin score (MRSS) at Week 48 as the primary outcome. We point out, however, that in the original study a mixed model for repeated measurements has been used to leverage all available information on the endpoint that has been collected at six visits in total. In this example, we only consider the final outcome data at Week 48. The distributions of the corresponding differences from baseline for each group are shown in Figure S4. There, we can notice a small difference between the tocilizumab group and the concurrent control arm, potentially indicating a treatment effect. At the same time, the difference between the current and historical control group appears to be even larger in favor of the concurrent controls. This suggests that caution is warranted when incorporating this external data source, as doing so could substantially impact the conclusions. Concerning baseline covariates that could be prognostic for this endpoint and explain some of the observed outcome differences we consider the baseline measurements of MRSS, the disease duration in days, C‐reactive protein (mg/L, log scale), the erythrocyte sedimentation (Sed) rate, the number of platelets, age, as well as sex and smoking behavior (never, previous, and current). For some of these variables the univariate distributions are depicted in Figure S5 grouped by the different treatments and studies. We can clearly see distributional differences between the current and historical controls w.r.t. these covariates. Yet, this does not automatically imply that these differences can also explain the observed variations between the respective outcome distributions. Figure S6 displays scatter plots of the outcome against the different baseline covariates. To further assist the assessment of the respective associations we add penalized splines fits for each (pooled) treatment group. We can see that, especially for the placebo group, the relevance of these baseline covariates for the endpoint of interest is not obvious in this example. Regarding the baseline MRSS and disease duration, we cannot infer a meaningful association with the outcome. Only for the C‐reactive protein (mg/L) we can observe a trend that is also consistent with the analogous results for the tocilizumab group. For the remainder of this illustrative example we keep using all of these covariates. Still, these exploratory analyses suggest that in real applications a careful selection of relevant covariates to be used by covariate‐adjusted BDB methods is important in order to provide useful information rather than adding noise.

The results of applying the different covariate‐adjusted BDB and further benchmark methods for estimating the treatment effect in this example are shown in Figure 8. For the current trial data we obtain a point estimate of a 1.53 unit difference between the MRSS change at Week 48 from baseline of the tocilizumab and placebo arm. However, from the corresponding 95% confidence interval we can deduce that this effect estimate is not significantly different from the null effect. When we naively pool the current and historical control group data the conclusion changes. Now, the magnitude of the effect estimate increases from 1.53 to 2.21 while in addition the width of the confidence interval shrinks by 0.39 units. As opposed to the current trial data analysis, we would then conclude that the estimated treatment effect is statistically significant at the two‐sided 5% level. Using dynamic borrowing methods can potentially alleviate this drastic change of conclusions. When applying MEM or PS‐MEM the results do not change much compared to the current data analysis. There is only a small difference in the point estimate and some decrease in the widths of the credible intervals. Both methods have an effective historical sample size of only one patient (rounded). Thus, the effect of PS weighting for this method is also limited in this example. There are two ways in which we can interpret these findings. On the one hand, we may conclude that PS‐MEM does a good job here in correctly gauging the historical control group as non‐exchangeable with the concurrent one, thereby prohibiting borrowing. On the other hand, it is equally possible that the observed behavior simply reflects our observation from the simulation study that PS‐MEM tends to not borrow much information in general. Turning to the class of commensurate priors, the variability in the results is more pronounced. With both, the PS weighted and unweighted version, we can depict a point estimate that is more shifted toward the pooled data estimate. The shift is slightly larger for the unweighted commensurate prior than for the weighted one. However, PS‐Commensurate has noticeably shorter credible intervals than the unweighted commensurate prior model. Indeed, this can potentially be attributed to the PS weighting being applied. In this example, there are a few historical control observations with very large PS weights (16.0, 11.5, and 7.6) possibly being the main cause for these differences. Although not a new insight, this example underpins the potential value of considering robust alternatives to classical PS weighting that prohibit such extreme weights, particularly in the context of rare disease trials with small sample sizes. Interestingly, BART seems to work better in this example compared to what we have seen before. Hence, based on the CI widths of the different versions of BART included in this comparison it appears like BART increases precision not only by adjusting for prognostic baseline covariates but also by borrowing some information from the historical control data. At the same time, the point estimate of the treatment effect is closely aligned with that of the current data analysis. The difficulty that remains in this regard is to better interpret how much and which of the historical information BART has borrowed. Nevertheless, these results give rise to keep considering BART as a borrowing method and try to understand when and why it may work or not.

**FIGURE 8:**
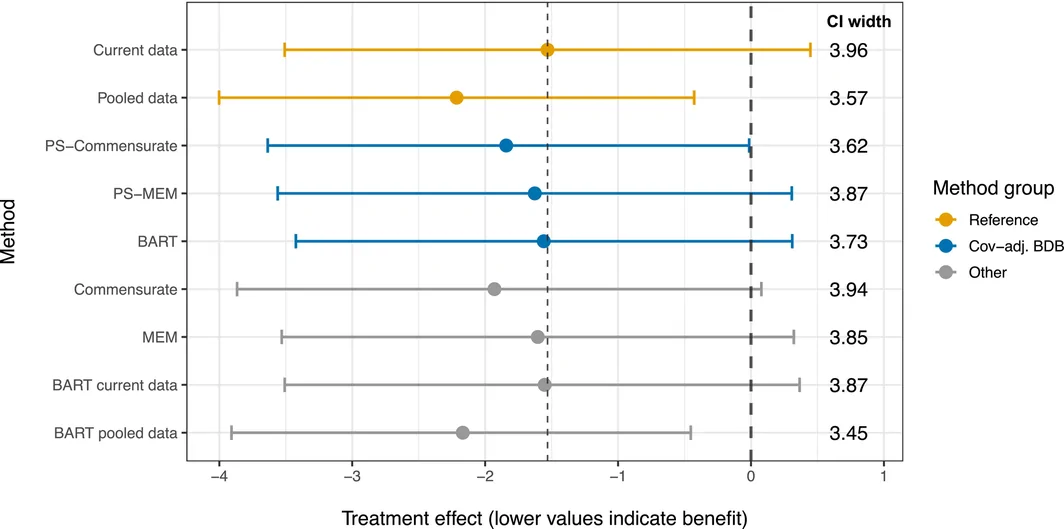
Point estimates and 95%‐confidence/credible intervals of different methods applied to the anonymized systemic sclerosis data. The thick dashed line references the null effect and the thin dashed line references the estimated effect using the current trial data only.

## 6 Discussion

Integrating historical control data in RCTs has become a research area of high interest in biostatistics. In this regard, Bayesian dynamic borrowing methods are relatively well established and understood. In recent years, there has been a rise in new method proposals suggesting to also incorporate baseline covariate information when borrowing historical control information by merging Bayesian methods with causal inference approaches. For these methods, overviews and systematic comparisons providing guidance are relatively scarce, making it difficult to opt for a specific approach in a practical context. We have addressed this issue in the present work by comparing three different methods of this broad class by means of simulations and two real data examples. Importantly, our comparison study is neutral, that is, we have not compared methods that we have introduced or modified ourselves. To conclude our study, we provide recommendations for which methods to use, discuss limitations of our work, and raise ideas for future research in this area.

### 6.1 Summary and Recommendations

In conclusion, among the three approaches evaluated, we recommend the PS‐Commensurate prior model. Although the known risks of information borrowing methods were more pronounced for PS‐Commensurate, we also found its behavior to be more predictable compared to the other methods, thereby making it easier to manage these risks. For instance, if there is higher uncertainty regarding the compatibility of the historical data, the hyperprior could be adjusted accordingly. PS‐MEM and BART, on the other hand, rely on different modeling mechanisms for information borrowing whose properties appeared to be more contingent upon nuisance factors in partly unclear ways. Consequently, we advocate to use informative prior distribution models more generally compared to alternative approaches for information borrowing. When knowledge about important prognostic baseline covariates is available these methods can be augmented with PS weighting to attain additional robustness. Nevertheless, we also want to highlight useful features of PS‐MEM and BART that future methodological developments of BDB methods could draw inspiration from. For instance, we find PS‐MEM to be particularly interpretable when it comes to the amount and the corresponding source of information it has borrowed information from. BART, on the other hand, underscores the potentials of prognostic covariate adjustment and proves advantageous for scenarios including potentially complex functional relationships with the outcome variable. Integrating these aspects could make dynamic borrowing methods more trustworthy and efficient.

### 6.2 Limitations of Our Work

Setting the goal of conducting an extensive neutral comparison study and, in the ideal case, making conclusive statements necessarily comes with certain limitations of the corresponding results. First, we only considered continuous outcomes with an assumed Gaussian distribution in our study but other endpoints such as counts or binary ones are also common in clinical trials. To what extent our findings also apply to these types of outcomes is unclear and warrants additional investigations. Other common data structures such as time‐to‐event or longitudinal data pose unique challenges and may even require extending the methods discussed herein. Two of the three methods included in our comparison would also allow to borrow information from more than one external source, which is a scenario we have not investigated. More generally, simulation studies will always be subject to criticism because of potentially interesting scenarios that have not been covered. For instance, studying the impact of further (nuisance) parameters like varying prognostic strengths of the baseline covariates could be of interest. Further, we decided against a more complicated and perhaps more realistic outcome‐generating simulation model in favor of being able to accurately quantify the impact of varying the different parameter values. Lastly, despite of our extensive literature review on covariate‐adjusted BDB methods, we want to acknowledge the possibility that we have missed on other promising methods that may perform equally well or even better than the ones included in our study. This issue will likely remain considering the sheer amount of already existing approaches and given that this research area remains active.

### 6.3 Future Research Directions

Obviously, future research could be geared toward improving or extending covariate‐adjusted BDB methods like the ones considered in this paper. In this regard, we think it is important to clearly communicate which shortcomings of already existing methods should be addressed such that method development does not become an end in itself. As an example, we think that the extension and application of dynamic borrowing methods to settings with time‐to‐event [41] or longitudinal data [42] still bears a lot of potential. Furthermore, and more closely related to our own contribution, we think that the evaluation of covariate‐adjusted BDB methods constitutes an issue on its own and demands more attention. This could, for instance, be addressed by publishing detailed case studies applying these methods to real data examples in rare disease indications. One rationale for this would be to demonstrate the actual benefits—or the absence thereof—of incorporating baseline covariate information in dynamic borrowing methods when it comes to practical applications. Such studies could perhaps also reveal “pitfalls” [11] of covariate‐adjusted BDB methods that were unknown before. Finally, real data examples could also inform the development of realistic simulation models for evaluating covariate‐adjusted BDB methods that are not overly complex but more involved than ours.

## Funding

This work was funded (or co‐funded) by the European Union's Horizon Europe Framework programme under grant agreement 101136365 (INVENTS), co‐funded by the Swiss State Secretariat for Education, Research and Innovation (SERI) and co‐funded by the UKRI Innovative UK under their Horizon Europe Guarantee scheme. Views and opinions expressed are those of the author(s) only and do not necessarily reflect those of the European Union or the European Health and Digital Executive Agency. Neither the European Union nor the granting authority can be held responsible for them.

## Conflicts of Interest

D.J., D.U., M.C.E. and F.M. are employees and own stocks of F. Hoffmann‐La Roche AG. T.F. declares no conflicts of interest.

## Supporting information


**Figure S1:** Additional simulation results for linear scenario.
**Figure S2:** Exponential decay function used for nonlinear simulation scenarios.
**Figure S3:** Additional simulation results for nonlinear scenario.
**Figure S4:** Distribution of the primary outcome measure (change in MRSS at Week 48 from baseline) across different treatments and studies based on anonymized data.
**Figure S5:** Summary of baseline characteristics of different treatment groups based on anonymized data.
**Figure S6:** Empirical associations between baseline variables and primary outcome measure based on anonymized data. The dashed lines are individual penalized spline fits of the respective effects using the pooled data.

## Data Availability

The data that support the findings of this study are available on request from the corresponding author. The data are not publicly available due to privacy or ethical restrictions.

## ENDNOTES

^1^
We omit the subscript denoting individual observations for notational convenience.

## References

[1] K. Viele , S. Berry , B. Neuenschwander , et al., “Use of Historical Control Data for Assessing Treatment Effects in Clinical Trials,” Pharmaceutical Statistics 13, no. 1 (2014): 41–54, 10.1002/pst.1589. PMC3951812 23913901

[2] M.‐H. Chen and J. G. Ibrahim , “Power Prior Distributions for Regression Models,” Statistical Science 15, no. 1 (2000): 46–60, 10.1214/ss/1009212673.

[3] B. Neuenschwander , G. Capkun‐Niggli , M. Branson , and D. J. Spiegelhalter , “Summarizing Historical Information on Controls in Clinical Trials,” Clinical Trials 7, no. 1 (2010): 5–18, 10.1177/1740774509356002. 20156954

[4] A. Kopp‐Schneider , S. Calderazzo , and M. Wiesenfarth , “Power Gains by Using External Information in Clinical Trials Are Typically Not Possible When Requiring Strict Type I Error Control,” Biometrical Journal 62, no. 2 (2020): 361–374, 10.1002/bimj.201800395. PMC7079072 31265159

[5] B. Neuenschwander , M. Branson , and D. J. Spiegelhalter , “A Note on the Power Prior,” Statistics in Medicine 28, no. 28 (2009): 3562–3566, 10.1002/sim.3722. 19735071

[6] H. Schmidli , S. Gsteiger , S. Roychoudhury , A. O'Hagan , D. Spiegelhalter , and B. Neuenschwander , “Robust Meta‐Analytic‐Predictive Priors in Clinical Trials With Historical Control Information,” Biometrics 70, no. 4 (2014): 1023–1032, 10.1111/biom.12242. 25355546

[7] S. J. Pocock , “The Combination of Randomized and Historical Controls in Clinical Trials,” Journal of Chronic Diseases 29, no. 3 (1976): 175–188, 10.1016/0021-9681(76)90044-8. 770493

[8] X. Wang , L. Suttner , T. Jemielita , and X. Li , “Propensity Score‐Integrated Bayesian Prior Approaches for Augmented Control Designs: A Simulation Study,” Journal of Biopharmaceutical Statistics 32, no. 1 (2022): 170–190, 10.1080/10543406.2021.2011743. 34939894

[9] P. R. Rosenbaum and D. B. Rubin , “The Central Role of the Propensity Score in Observational Studies for Causal Effects,” Biometrika 70, no. 1 (1983): 41–55, 10.1093/biomet/70.1.41.

[10] A.‐L. Boulesteix , H. Binder , M. Abrahamowicz , W. Sauerbrei , and and for the Simulation Panel of the STRATOS Initiative , “On the Necessity and Design of Studies Comparing Statistical Methods,” Biometrical Journal 60, no. 1 (2018): 216–218, 10.1002/bimj.201700129. 29193206

[11] A.‐L. Boulesteix , S. Lauer , and M. J. A. Eugster , “A Plea for Neutral Comparison Studies in Computational Sciences,” PLoS One 8, no. 4 (2013): e61562, 10.1371/journal.pone.0061562. PMC3634809 23637855

[12] W. Wei , Y. Zhang , S. Roychoudhury , and Alzheimer's Disease Neuroimaging Initiative , “Propensity Score Weighted Multi‐Source Exchangeability Models for Incorporating External Control Data in Randomized Clinical Trials,” Statistics in Medicine 43, no. 20 (2024): 3815–3829, 10.1002/sim.10158. 38924575

[13] T. Zhou and Y. Ji , “Incorporating External Data Into the Analysis of Clinical Trials via Bayesian Additive Regression Trees,” Statistics in Medicine 40, no. 28 (2021): 6421–6442, 10.1002/sim.9191. 34494288

[14] D. B. Rubin , “Estimating Causal Effects of Treatments in Randomized and Nonrandomized Studies,” Journal of Educational Psychology 66, no. 5 (1974): 688–701, 10.1037/h0037350.

[15] M. Valancius , H. Pang , J. Zhu , S. R. Cole , M. Jonsson Funk , and M. R. Kosorok , “A Causal Inference Framework for Leveraging External Controls in Hybrid Trials,” Biometrics 80, no. 4 (2024): ujae095, 10.1093/biomtc/ujae095. PMC11546536 39514601

[16] X. Zhou , J. Zhu , C. Drake , and H. Pang , “Causal Estimators for Incorporating External Controls in Randomized Trials With Longitudinal Outcomes,” Journal of the Royal Statistical Society Series A: Statistics in Society 188, no. 3 (2025): 791–818, 10.1093/jrsssa/qnae075.

[17] A. Oganisian and J. A. Roy , “A Practical Introduction to Bayesian Estimation of Causal Effects: Parametric and Nonparametric Approaches,” Statistics in Medicine 40, no. 2 (2021): 518–551, 10.1002/sim.8761. PMC8640942 33015870

[18] S. Senn , “Causal Estimates for External Controls. How Reasonable Are the Assumptions?,” Journal of the Royal Statistical Society Series A: Statistics in Society 189, no. 1 (2026): 607–610, 10.1093/jrsssa/qnaf127.

[19] B. P. Hobbs , B. P. Carlin , S. J. Mandrekar , and D. J. Sargent , “Hierarchical Commensurate and Power Prior Models for Adaptive Incorporation of Historical Information in Clinical Trials,” Biometrics 67, no. 3 (2011): 1047–1056, 10.1111/j.1541-0420.2011.01564.x. PMC3134568 21361892

[20] C. Wang , H. Li , W.‐C. Chen , et al., “Propensity Score‐Integrated Power Prior Approach for Incorporating Real‐World Evidence in Single‐Arm Clinical Studies,” Journal of Biopharmaceutical Statistics 29, no. 5 (2019): 731–748, 10.1080/10543406.2019.1657133. 31530111

[21] M. Liu , V. Bunn , B. Hupf , J. Lin , and J. Lin , “Propensity‐Score‐Based Meta‐Analytic Predictive Prior for Incorporating Real‐World and Historical Data,” Statistics in Medicine 40, no. 22 (2021): 4794–4808, 10.1002/sim.9095. 34126656

[22] C. J. Lewis , S. Sarkar , J. Zhu , and B. P. Carlin , “Borrowing From Historical Control Data in Cancer Drug Development: A Cautionary Tale and Practical Guidelines,” Statistics in Biopharmaceutical Research 11, no. 1 (2019): 67–78, 10.1080/19466315.2018.1497533. PMC6703839 31435458

[23] F. Li , P. Ding , and F. Mealli , “Bayesian Causal Inference: A Critical Review,” Philosophical Transactions of the Royal Society A: Mathematical, Physical and Engineering Sciences 381, no. 2247 (2023): 20220153, 10.1098/rsta.2022.0153. 36970828

[24] E. M. Alt , X. Chang , X. Jiang , et al., “LEAP: The Latent Exchangeability Prior for Borrowing Information From Historical Data,” Biometrics 80, no. 3 (2024): ujae083, 10.1093/biomtc/ujae083. 39329230

[25] B. P. Hobbs , D. J. Sargent , and B. P. Carlin , “Commensurate Priors for Incorporating Historical Information in Clinical Trials Using General and Generalized Linear Models,” Bayesian Analysis 7, no. 3 (2012): 639–674, 10.1214/12-BA722. PMC4007051 24795786

[26] J. Huang , F. Jia , J. Li , et al., “Dynamic Information Borrowing From External Data in Clinical Trials: The Elastic Commensurate Prior Approach,” Statistics in Medicine 44, no. 13–14 (2025): e70129, 10.1002/sim.70129. 40468849

[27] A. M. Kaizer , J. S. Koopmeiners , and B. P. Hobbs , “Bayesian Hierarchical Modeling Based on Multisource Exchangeability,” Biostatistics 19, no. 2 (2018): 169–184, 10.1093/biostatistics/kxx031. PMC5862286 29036300

[28] H. A. Chipman , E. I. George , and R. E. McCulloch , “BART: Bayesian Additive Regression Trees,” Annals of Applied Statistics 4, no. 1 (2010): 266–298, 10.1214/09-AOAS285.

[29] J. L. Hill , “Bayesian Nonparametric Modeling for Causal Inference,” Journal of Computational and Graphical Statistics 20, no. 1 (2011): 217–240, 10.1198/jcgs.2010.08162.

[30] D. B. Rubin , “The Bayesian Bootstrap,” Annals of Statistics 9, no. 1 (1981): 130–134, 10.1214/aos/1176345338.

[31] T. P. Morris , I. R. White , and M. J. Crowther , “Using Simulation Studies to Evaluate Statistical Methods,” Statistics in Medicine 38, no. 11 (2019): 2074–2102, 10.1002/sim.8086. PMC6492164 30652356

[32] R Core Team , R: A Language and Environment for Statistical Computing (R Foundation for Statistical Computing, 2025).

[33] M. Secrest and I. Gravestock , “psborrow2: Bayesian Dynamic Borrowing Analysis and Simulation,” (2026), R package version 0.0.5.1, https://github.com/Genentech/psborrow2.

[34] J. Gabry , R. Češnovar , A. Johnson , and S. Bronder , “cmdstanr: R Interface to CmdStan,” (2025), R package version 0.9.0, https://mc‐stan.org/cmdstanr/.

[35] V. Dorie , H. Chipman , and R. McCulloch , “dbarts: Discrete Bayesian Additive Regression Trees Sampler,” (2026), R package version 0.9–33, https://github.com/vdorie/dbarts.

[36] K. Ushey and H. Wickham , “renv: Project Environments,” (2026), R package version 1.2.3, https://rstudio.github.io/renv/.

[37] F. Li , L. E. Thomas , and F. Li , “Addressing Extreme Propensity Scores via the Overlap Weights,” American Journal of Epidemiology 188, no. 1 (2019): 250–257, 10.1093/aje/kwy201. 30189042

[38] S. Friedrich and T. Friede , “On the Role of Benchmarking Data Sets and Simulations in Method Comparison Studies,” Biometrical Journal 66, no. 1 (2024): 2200212, 10.1002/bimj.202200212. 36810737

[39] Y. Yao , A. Vehtari , D. Simpson , and A. Gelman , “Using Stacking to Average Bayesian Predictive Distributions (With Discussion),” Bayesian Analysis 13, no. 3 (2018): 917–1007, 10.1214/17-BA1091.

[40] C. P. Denton and D. Khanna , “Systemic Sclerosis,” Lancet 390, no. 10103 (2017): 1685–1699, 10.1016/S0140-6736(17)30933-9. 28413064

[41] E. Kwiatkowski , J. Zhu , X. Li , H. Pang , G. Lieberman , and M. A. Psioda , “Case Weighted Power Priors for Hybrid Control Analyses With Time‐To‐Event Data,” Biometrics 80, no. 2 (2024): ujae019, 10.1093/biomtc/ujae019. PMC10968526 38536747

[42] H. Qi , D. Rizopoulos , E. Lesaffre , and J. van Rosmalen , “Incorporating Historical Controls in Clinical Trials With Longitudinal Outcomes Using the Modified Power Prior,” Pharmaceutical Statistics 21, no. 5 (2022): 818–834, 10.1002/pst.2195. PMC9356117 35128780

