# Supplementary material for: Bayesian Methods Incorporating Baseline Covariates for Borrowing Historical Control Data in Randomized Clinical Trials: A Neutral Comparison Study
Source: Stat Med. 2026 Oct 22;45(25-27):e70785. doi: 10.1002/sim.70785 (PMC13690490; doi:10.1002/sim.70785)
Supplement: Supplementary file 1 [file SIM-45-0-s001.pdf]

Supplement to "Bayesian Methods Incorporating Baseline Covariates for Borrowing Historical Control Data in Randomised Clinical Trials: A Neutral Comparison Study"

David Jesse[1,2], Diane Uschner[1], Markus Elze[1], Tim Friede[2,3], Francois Mercier[1]

[1] F. Hoffmann-La Roche AG, Basel, Switzerland

[2] Department of Medical Statistics, University Medical Center Göttingen, Göttingen, Germany

[3] DZKJ (German Center for Child and Adolescent Health), Göttingen, Germany

S.1 Additional Simulations

This section provides two supplemental simulation studies for obtaining further insights beyond the results presented in the main manuscript.

S.1.1 Direct Comparison of Borrowing and No-Borrowing BART

In the first study, we used the same data-generating model as presented in Section 4.1.2 in the main text. We only considered scenarios with perfect exchangeability, i.e. where both $\delta_u = 0$ and $\delta_2 = 0$ (as well as $\delta_3 = 0$) hold.

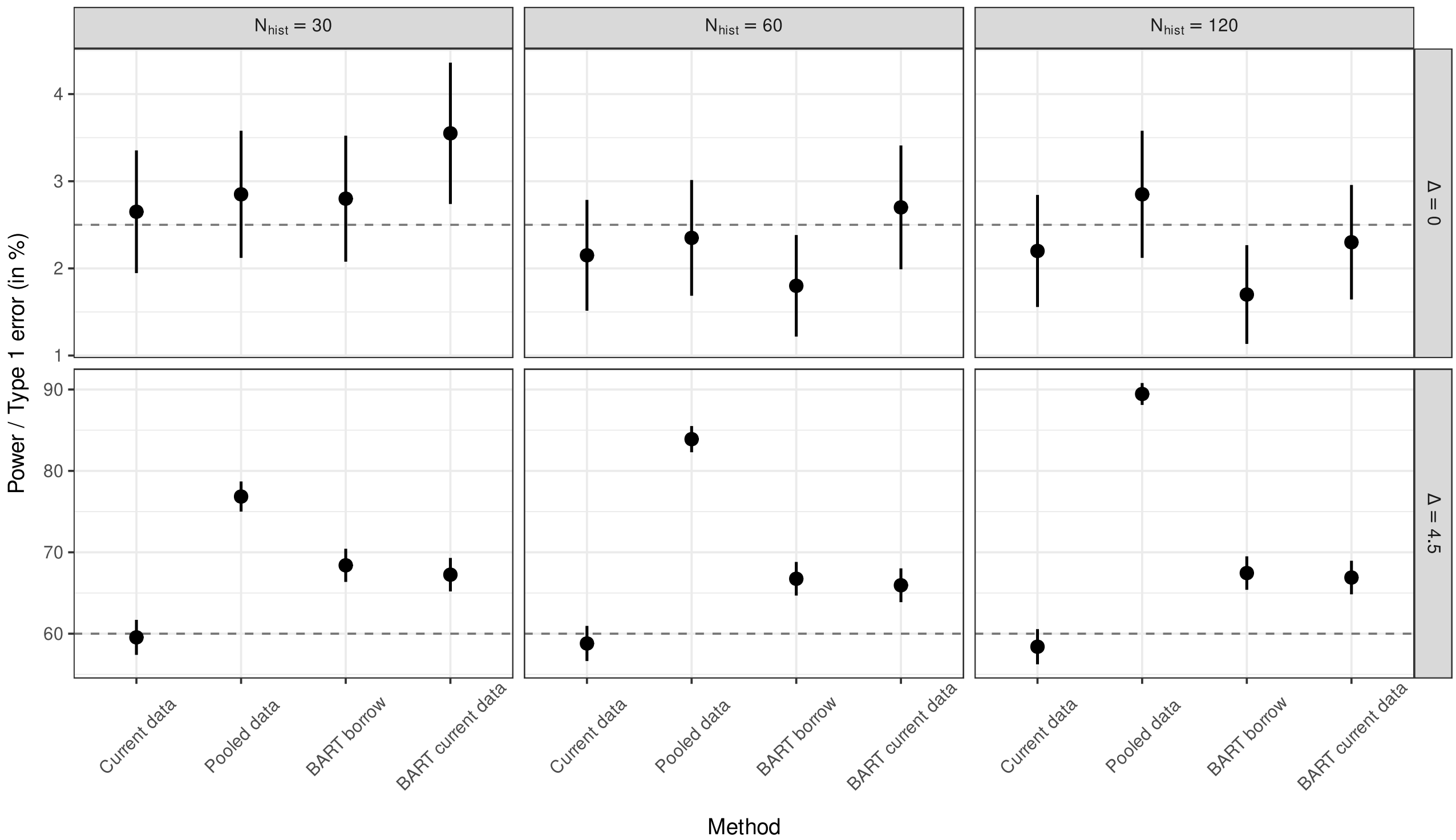

Figure S1: Additional simulation results for linear scenario.

S.1.2 Nonlinear Data-generating Mechanism

In the second study, we modified the data-generating mechanism to include a nonlinear predictor. Specifically, we replaced $\beta_2 x_2$ with an exponential decay function $b_1 \exp(-b_2 x_2)$. The covariates are now generated as follows:

- $x_1 \sim \mathcal{N}(6, 8)$
- $x_2 \sim \text{Unif }(0, 10)$
- $x_3 \sim \mathcal{N}(0, 6)$

The exponential decay function is parametrised with $b_1 = 12$ and $b_2 = 0.6$. With the distribution of x_2 this predictor term has mean 10 and variance 8.02. Thus, the marginal properties of the main simulation model are nearly retained. Below is a plot of the exponential decay function.

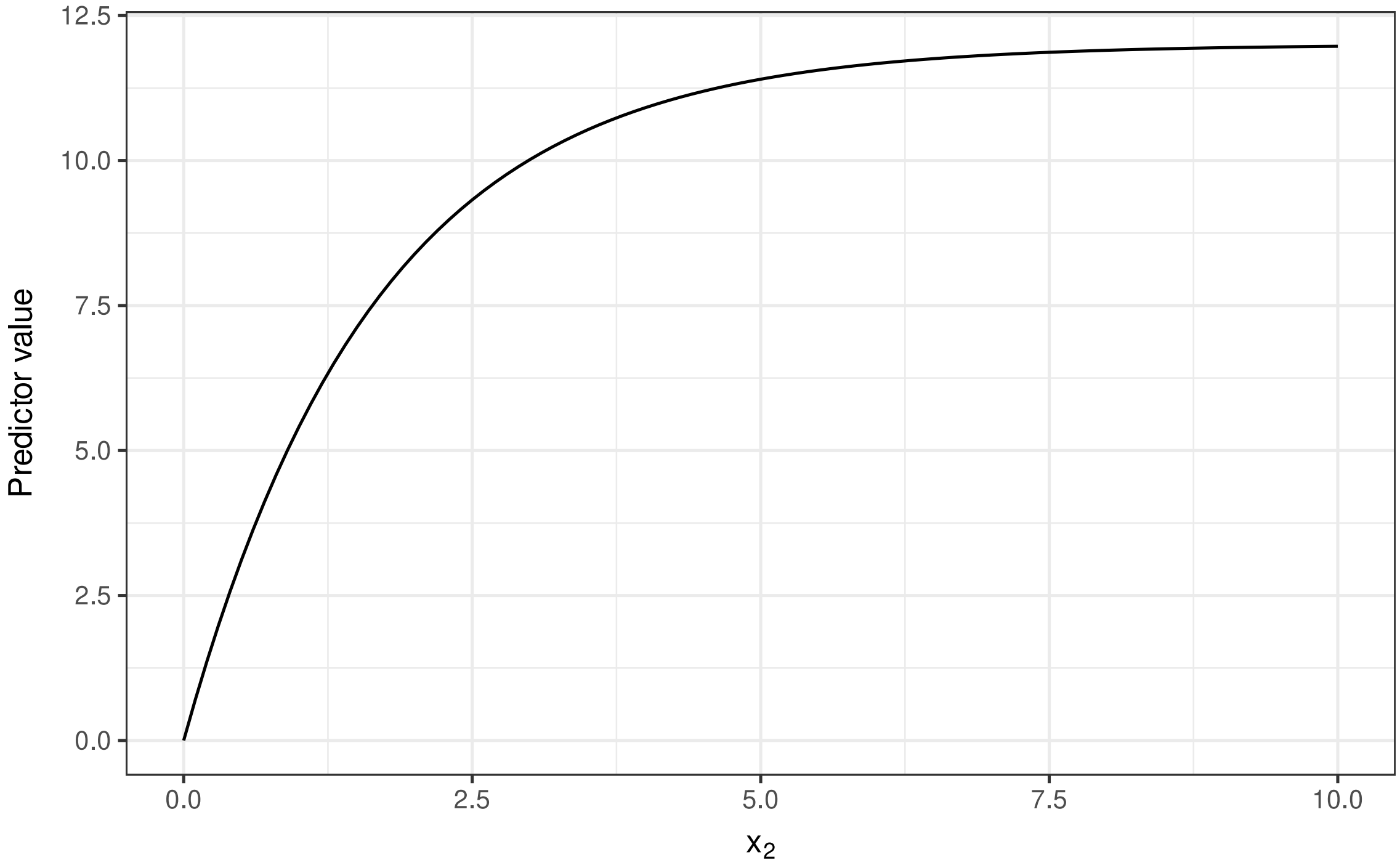

Figure S2: Exponential decay function used for nonlinear simulation scenarios.

Again, we only consider scenarios with perfect exchangeability for these simulations. These additional simulations were primarily motivated by the BART method and by the question whether its borrowing behaviour would differ if a nonlinear predictor term is included in the data-generating mechanism. Since this simulation study uses a data-generating model that is different from the one in the main manuscript we also included the other borrowing methods in this comparison.

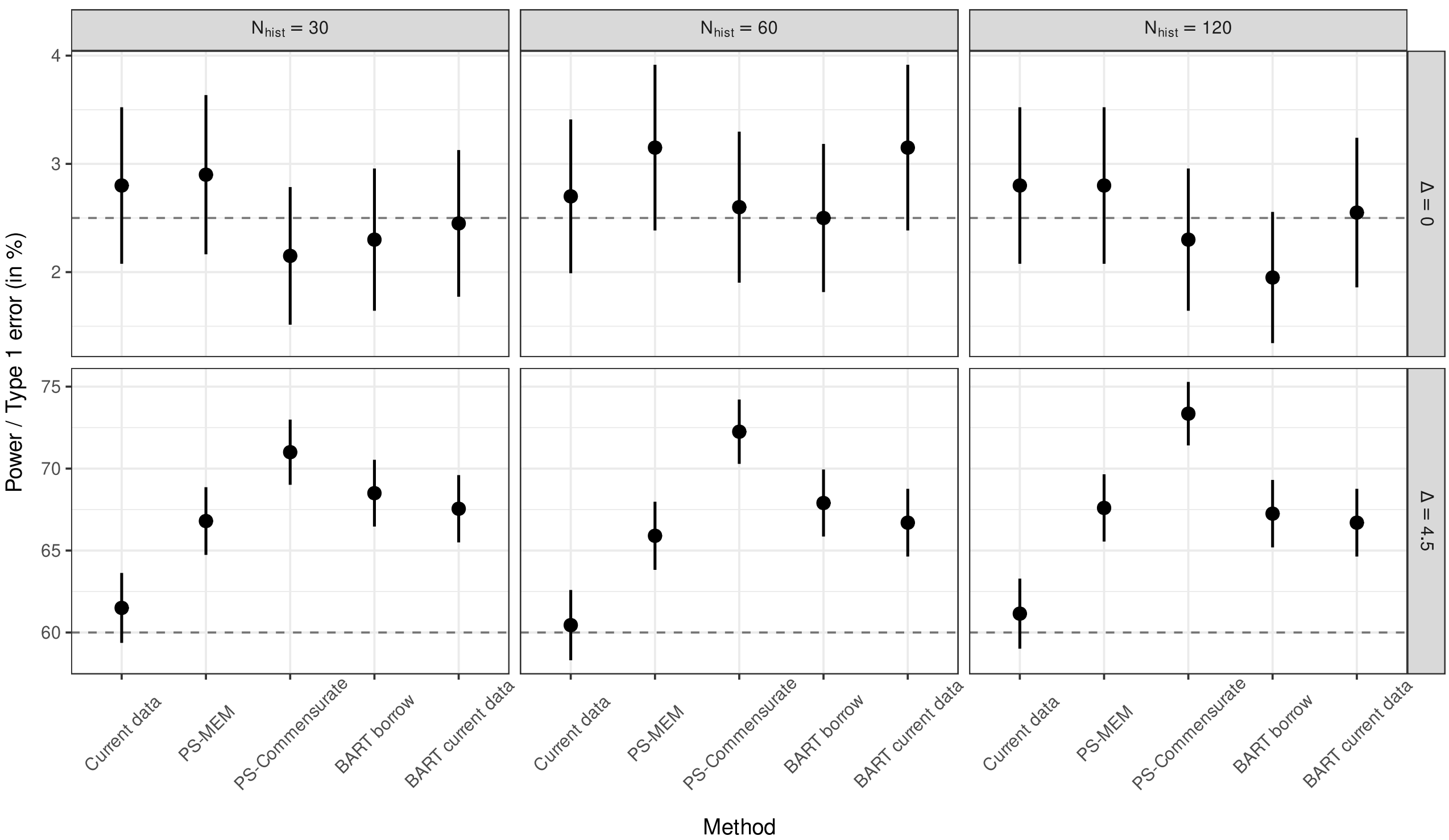

Figure S3: Additional simulation results for nonlinear scenario.

S.2 Exploratory Data Analyses of the Systemic Sclerosis Trial Data

This section contains supplementary exploratory data analyses of the anonymised systemic sclerosis trial example that are referenced in section 5.2 of the main manuscript.

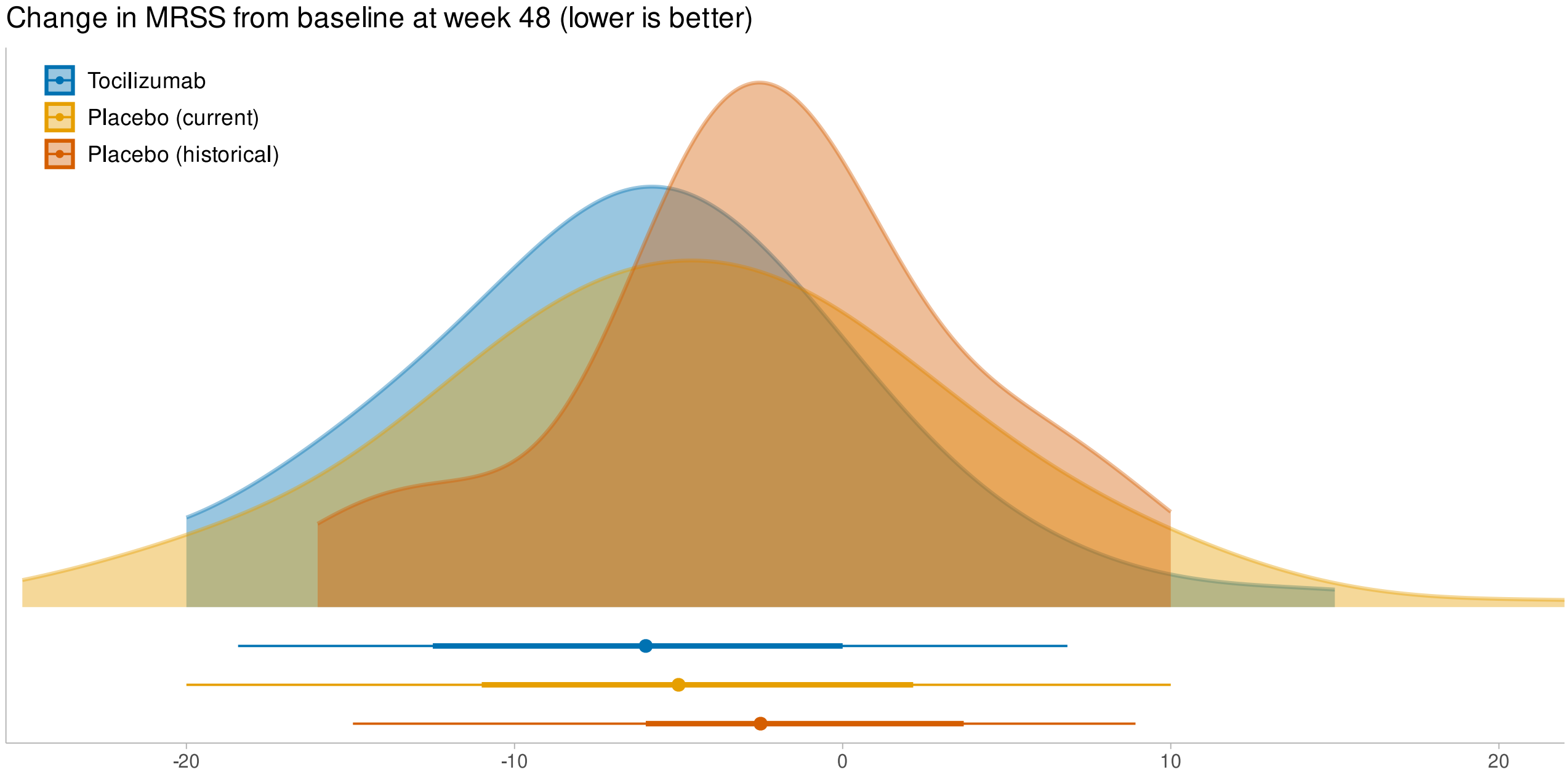

Figure S4: Distribution of the primary outcome measure (change in MRSS at week 48 from baseline) across different treatments and studies based on anonymised data.

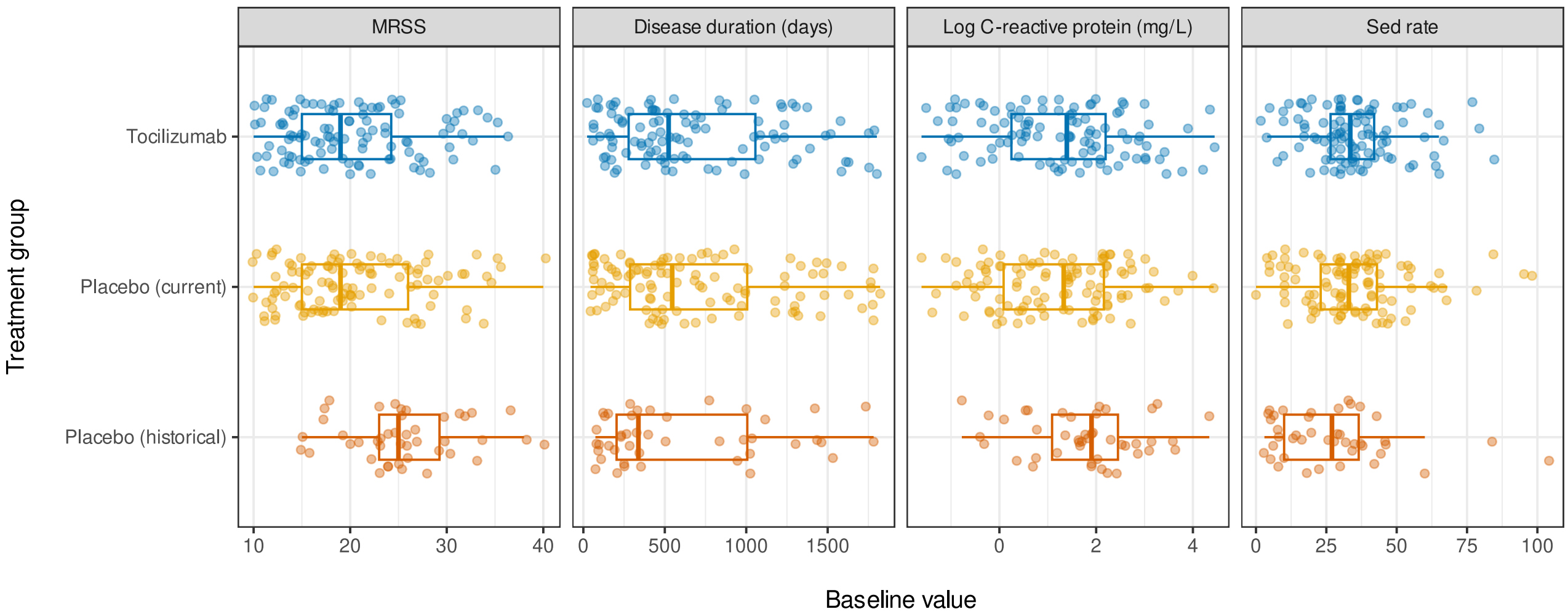

Figure S5: Summary of baseline characteristics of different treatment groups based on anonymised data.

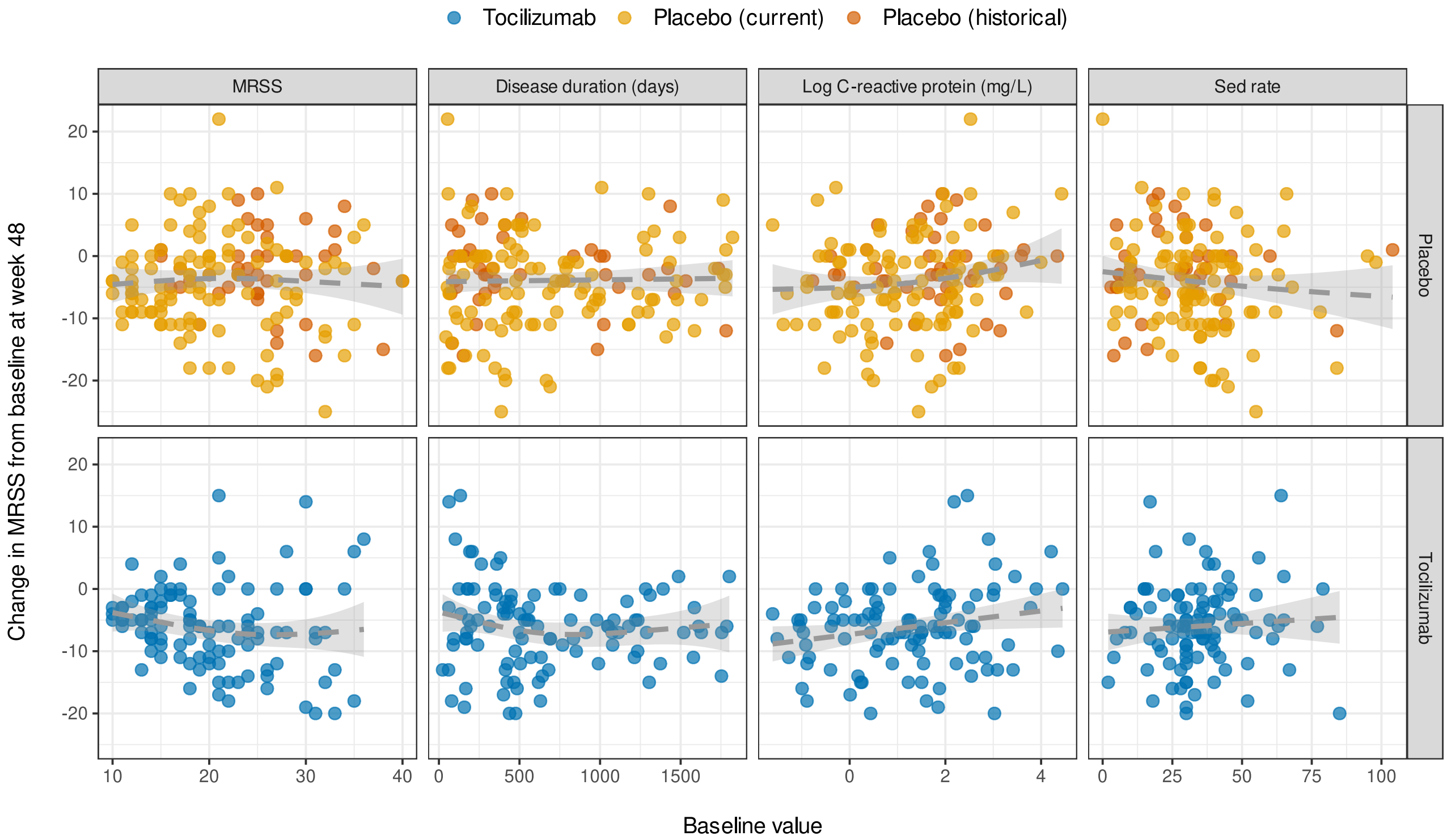

Figure S6: Empirical associations between baseline variables and primary outcome measure based on anonymised data. The dashed lines are individual penalized spline fits of the respective effects using the pooled data.

S.3 Impact of the Outcome Scale on Borrowing Behaviour of PS-MEM

Here, we present a small investigation of how the borrowing behaviour (strength of historical information borrowing) of PS-MEM depends on the scale of the outcome for Gaussian data. We start by simulating a single data set from our simulation model with perfect exchangeability, i.e. where the current and historical control groups have identical distributions. With this parametrisation the control groups have a marginal mean of 18 and a marginal variance of 81.

```
box::use(
  borrow/ps_mem[borrow_ps_mem],
  simfuns/gen_data[Simulator]
)

set.seed(42)

# Initialize and parametrize data simulation model (perfect exchangeability)
simulator = Simulator$new(
  shift_x2 = 0,
  shift_x3 = 0,
  shift_u = 0,
  n_trt = 60,
  n_ctrl = 30,
  n_hist = 60,
  b_trt = 0
)

# Simulate one "raw" data set
dt = simulator$gen_data()
```

In the subsequent step we apply the PS-MEM method to this data set but consider different scalings of the outcome. There, it can be seen how the amount of historical information that gets borrowed (quantified by the posterior exchangeability probability or the effective historical sample size) depends on the scale of the outcome.

```
# Different scalings for the outcome
scalings = c(0.01, 0.05, 0.1, 0.2, 0.5, 1, 2, 4, 5, 10)

# Apply PS-MEM to data set with different scalings
li_res = lapply(scalings, function(a) {
  new_dt = copy(dt)
  new_dt[, y := a * y]

  borrow_ps_mem(
    data = new_dt,
    outcome = "y", treatment = "trt", source = "source",
    covariates = paste0("x", 1:3),
    family = gaussian()
  )
})

# Collect/organize results
dt_res = data.table(
  scaling = scalings,
  post_exchange_prob = vapply(li_res, \(x) x$mem$exchange_probs[[2]], numeric(1)),
  ehss = vapply(li_res, \(x) x$mem$ess[[2]], numeric(1))
)

# Print results
kable(
  dt_res,
  digits = c(2, 2, 1),
  col.names = c(
    "Scaling",
    "Post. exchangeability prob.",
    "EHSS"
  )
)
```

Scaling	Post. exchangeability prob.	EHSS
0.01	0.93	28.0
0.05	0.74	22.1
0.10	0.58	17.5
0.20	0.41	12.3
0.50	0.22	6.5
1.00	0.12	3.7
2.00	0.07	2.0
4.00	0.03	1.0
5.00	0.03	0.8
10.00	0.01	0.4
